# Clustering and Negative Feedback by Endocytosis in Planar Cell Polarity Signaling Is Modulated by Ubiquitinylation of Prickle

**DOI:** 10.1371/journal.pgen.1005259

**Published:** 2015-05-21

**Authors:** Bomsoo Cho, Gandhy Pierre-Louis, Andreas Sagner, Suzanne Eaton, Jeffrey D. Axelrod

**Affiliations:** 1 Department of Pathology, Stanford University School of Medicine, Stanford, California, United States of America; 2 Department of Developmental Biology, Stanford University School of Medicine, Stanford, California, United States of America; 3 Max Planck Institute of Molecular Cell Biology and Genetics, Dresden, Germany; HHMI/Rutgers University, UNITED STATES

## Abstract

The core components of the planar cell polarity (PCP) signaling system, including both transmembrane and peripheral membrane associated proteins, form asymmetric complexes that bridge apical intercellular junctions. While these can assemble in either orientation, coordinated cell polarization requires the enrichment of complexes of a given orientation at specific junctions. This might occur by both positive and negative feedback between oppositely oriented complexes, and requires the peripheral membrane associated PCP components. However, the molecular mechanisms underlying feedback are not understood. We find that the E3 ubiquitin ligase complex Cullin1(Cul1)/SkpA/Supernumerary limbs(Slimb) regulates the stability of one of the peripheral membrane components, Prickle (Pk). Excess Pk disrupts PCP feedback and prevents asymmetry. We show that Pk participates in negative feedback by mediating internalization of PCP complexes containing the transmembrane components Van Gogh (Vang) and Flamingo (Fmi), and that internalization is activated by oppositely oriented complexes within clusters. Pk also participates in positive feedback through an unknown mechanism promoting clustering. Our results therefore identify a molecular mechanism underlying generation of asymmetry in PCP signaling.

## Author Summary

Many epithelial cells display a level of organization in which cellular structures or appendages are positioned asymmetrically within the cell along an axis perpendicular to the apical-basal axis of the cell. When the direction of this polarization is coordinated within the plane of the epithelium, this phenomenon is referred to as planar cell polarity (PCP). PCP is organized, at least in part, by a group of molecules that interact across cell-cell junctions and segregate into two groups that localize on opposite sides of each cell. Their asymmetric localization is thought to both produce molecular asymmetry, and to mark polarized domains within the cell for subsequent morphological polarization. In segregating to produce molecular asymmetry, these proteins participate in both positive and negative feedback, much like ferromagnets, to align their localization within and between neighboring cells. In this work, we identify a mechanism for negative feedback that utilizes the protein Prickle, one of the PCP signaling components. Levels of Prickle are precisely regulated, in part by a ubiquitinylation mechanism that targets excess protein for degradation. Prickle mediates internalization and removal of one class of PCP proteins, thereby causing repulsion of opposite ‘poles.’ Excess Prickle disrupts this mechanism and interferes with establishing polarity.

## Introduction

PCP is the tissue-level organization of cells in the plane of an epithelium, resulting from the coordinated acquisition of cellular polarity orthogonal to the apical-basal axis. PCP signaling controls the polarity of numerous epithelial cells in both *Drosophila* and vertebrates. In *Drosophila*, the most thoroughly studied planar polarized tissue is the fly wing, in which each cell produces a trichome (“hair”), that in wild type, emerges from the distal side of the cell and points distally [1]. PCP mutants cause disruption of this pattern. In vertebrates, many of the PCP signaling components identified in flies are conserved, and function together with additional regulators not present in flies. Defects in vertebrate PCP result in a range of developmental anomalies and diseases including open neural tube defects, conotruncal heart defects, and disruption of sensory hair cell polarity. PCP is also believed to underlie the directed migration of malignant cells during invasion and metastasis (reviewed in [2,3]). Despite increasing study, understanding of PCP signaling mechanisms remains limited.

A key feature of PCP signaling is the generation of subcellular asymmetry in which critical signaling components segregate to form complexes on opposite sides of the cell. Components of the *Drosophila* PCP signaling mechanism may be divided into three functional module types including a core module, global directional modules and a suite of tissue specific effector modules that execute morphological polarization in individual tissues [4]. The core module acts both to amplify asymmetry, and to coordinate polarization between neighboring cells, producing a local alignment of polarity. Proteins in the core signaling module, including the serpentine receptor Frizzled (Fz) [5,6], the multi-domain protein Dishevelled (Dsh) [7,8], the Ankryin repeat protein Diego (Dgo) [9], the 4-pass transmembrane protein Van Gogh (Vang; a.k.a. Strabismus) [10,11], the Lim domain protein Prickle (Pk) [12], the seven-transmembrane atypical cadherin Flamingo (Fmi; a.k.a. Starry night) [13,14], and perhaps others [15,16], adopt asymmetric subcellular localizations that predict the hair polarity pattern (reviewed in [17]). These proteins communicate at cell boundaries, recruiting one group to the distal side of cells, and the other to the proximal side, thereby aligning the polarity of adjacent cells [18,19].

Insight into this mechanism comes from studies of clones either not expressing or overexpressing core PCP components. These clones display characteristic perturbations (or lack thereof) of cells in nearby wing tissue (referred to as domineering non-autonomy) [5,10,20,21] that have been exploited in conjunction with mathematical modeling to better understand the signaling mechanisms (reviewed in [22]).

Several global modules have been proposed to provide tissue-level directional information to the core module, aligning polarization to the tissue axes. These include the Fat/Dachsous/Four-jointed module [23], Wnt4/Wg [24], and other undefined signals [25]. The Ft/Ds/Fj module is thought to orient core signaling by organizing polarized microtubule-dependent vesicular trafficking of distal core proteins [26–28].

An important unanswered question is how asymmetric subcellular localization and amplification of core components is achieved. It is proposed that an input bias from one or more of the global modules is amplified by feedback mechanisms, eventually producing strong asymmetric localization of core components. Theoretical models indicate that polarization requires a combination of a short range cooperative (positive) feedback and a long range inhibitory (negative) signal [29]. Polarization of isolated cells requires that the long range signal be intracellular and diffusible. However, coordinated polarization within sheets of cells allows the possibility that the long range negative signal, as well as the short range positive signal, might operate through contact-mediated intercellular mechanisms.

Cell biological and biophysical analyses have indicated that the transmembrane core components Fz, Fmi and Vang can assemble into stable intercellular complexes mediated by Fmi homodimerization and perhaps direct interaction between Fz and Vang ([Fz-Fmi]-[Fmi-Vang], where square brackets indicate complexed proteins in adjacent cell membranes) [30,31]. These complexes may be regulated by internalization and recycling [32]. Additional analyses suggest that the peripheral membrane associated core proteins Dsh, Pk and Dgo are required for amplification of core asymmetry [32,33]. These proteins induce stabilization of intercellular complexes and clustering into discrete puncta, of which presence and size correlate with amplification of asymmetry [32]. Clustering may be a mechanism for positive feedback. In addition, it has been proposed that oppositely oriented intercellular complexes antagonize each other, a process we refer to as mutual antagonism [18,19]. This competitive inhibition could be a form of negative long range intercellular feedback. Molecular mechanisms for these events are not known.

Regulation of levels of each of the core proteins is required for correct core PCP function, such that clonal loss or gain of function perturbs normal polarization (summarized in [18]). Ubiquitinylation has been identified as a regulator of core PCP function. E3 ligases covalently attach ubiquitin to target proteins to control protein levels and intracellular trafficking of membrane proteins [34,35]. Poly-ubiquitinylation often works as a proteasomal degradation signal, while mono-ubiquitinylation of membrane proteins typically drives targets toward vesicular trafficking pathways. Mammalian Prickle-like proteins undergo Dvl (Dsh)-dependent ubiquitinylation by Smurf E3 ubiquitin ligases and degradation at the cell surface that is required for cellular asymmetry and neural tube closure [36]. In *Drosophila*, the Cullin 3 (Cul3) E3 ligase complex regulates apicolateral Dsh levels, which in turn, regulates accumulation of other polarity proteins [37]. Disruption of this mechanism causes only mild PCP phenotypes. Recently, RNA interference (RNAi) of SkpA (a Cul1 complex component) was shown by both immunofluorescence and Western blot to produce accumulation of Pk in pupal wings [38]. No PCP phenotype was demonstrated or consequences characterized, nor was ubiquitinylation of Pk demonstrated.

To characterize ubiquitinylation pathways for PCP control, we carried out an RNAi screen for *Drosophila* E3 ligases and found Cul1 E3 ligase complex components as regulators of PCP in *Drosophila* wings. We provide evidence suggesting that the Cul1 complex directly regulates Pk levels and, indirectly, accumulation of other core PCP proteins. Blocking this mechanism disrupts asymmetric subcellular localization of core PCP proteins and correct hair orientation. Furthermore, our results lead us to describe a mechanism for feedback regulation required to generate asymmetry. We provide evidence for an intercellular long range inhibitory mechanism (mutual exclusion), in which Pk, triggered by Fz complexes, antagonizes the accumulation of Pk-Vang-Fmi complexes by promoting internalization. These results show that Cul1 complex-mediated control of Pk is required to ensure amplification and molecular polarization of the core module.

## Results

### Identification of the Cul1 E3 ligase complex as a regulator of trichome polarity patterning in the wing

**Fig 1:**
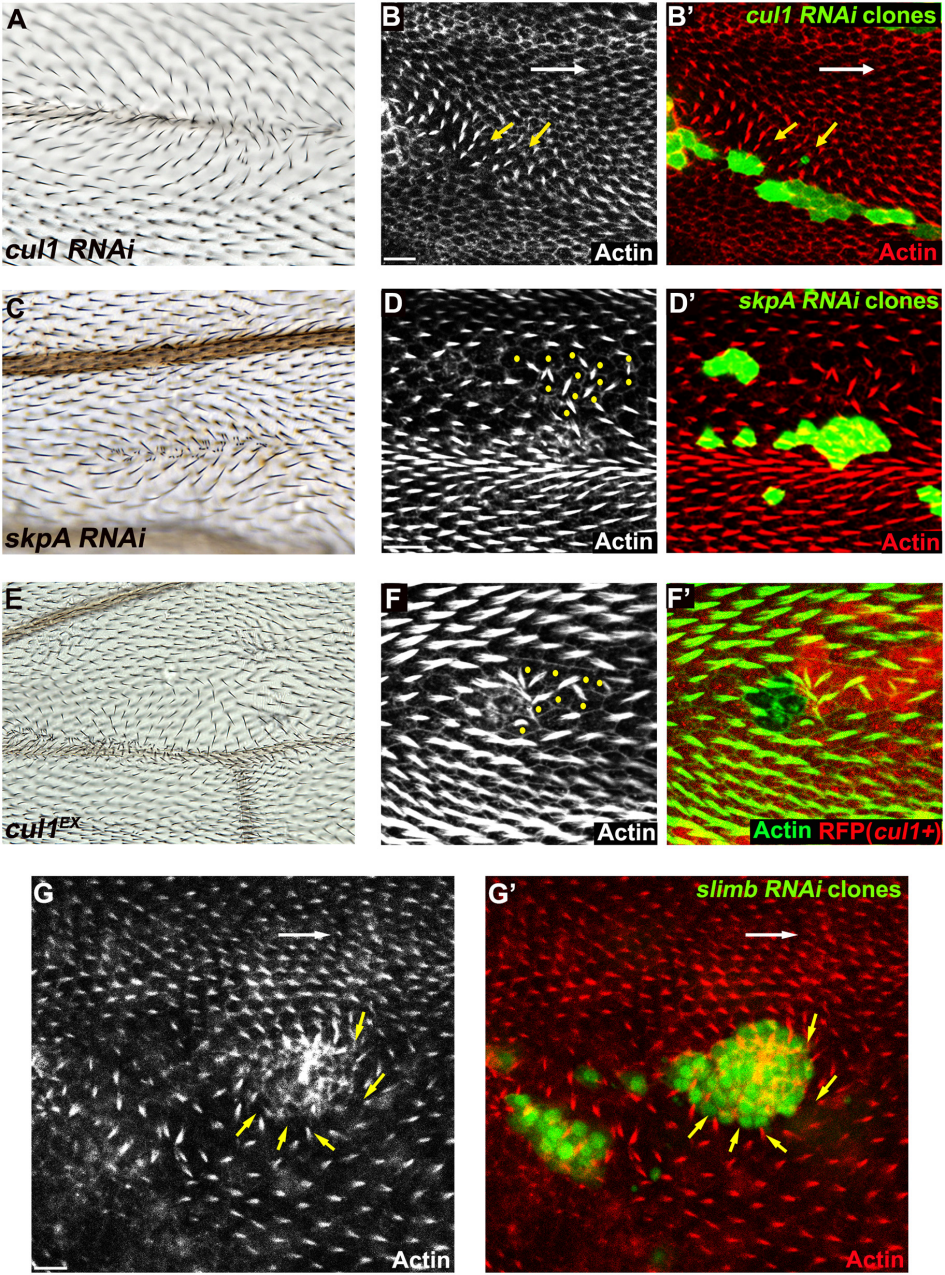
The Cul1 complex is required for hair and trichome polarity in the wing. Multiple hairs and swirling of hairs in adult wings (A, C, E) bearing *cul1* (A) and *skpA* (C) knock-down, and *cul1* null (*cul1* ^*EX*^) mutant (E) clones. Growth of pre-hairs in knock-down clones (labeled by GFP) of *cul1* (B), *skpA* (D), and *slimb* (G), and in *cul1* null mutant clones (F, no RFP expression), is delayed (absent or smaller trichomes). Polarity of pre-hairs in neighboring wild type cells is also affected (white arrow for normal pre-hair direction and yellow arrows for abnormal pre-hair direction in B and G. Yellow dots in D and F indicate wild-type cells with defects in pre-hair polarity). 32~34hr APF pupal wings were stained with phalloidin to label Actin-rich trichomes (red in B, D, and G; green in F). Scale bars: 10μm. Genotypes are (A, B) *y*, *w*, *hsflp/+(Y); UAS-cul1* ^*IR108558*^ */+; actP>CD2>GAL4*, *UAS-GFP/+*, (C, D) *y*, *w*, *hsflp/+(Y); UAS-skpA* ^*IR32789*^ */+; actP>CD2>GAL4*, *UAS-GFP/+*, (E, F) *y*, *w*, *hsflp/+(Y); FRT42D*, *cul1* ^*EX*^ */FRT42D*, *ubiP-NLS*::*mRFP*, (G) *y*, *w*, *hsflp/+(Y); +/+; actP>CD2>GAL4*, *UAS-GFP/UAS-slimb* ^*IRFBst0033898*^.

To identify ubiquitinylation pathways regulating PCP, RNAi constructs knocking down E3 ubiquitin ligases were screened for wing hair polarity defects. In a previous genome-wide in vivo RNAi screen, *pnr-GAL4* (and *MS1096GAL4*) driven expression of RNAi’s targeting several E3s showed notal bristle polarity defects suggesting possible PCP defects [39]. However, many E3 RNAi’s caused lethality, and for these polarity could not be assessed. To overcome this limitation, we re-examined E3 RNAi constructs that caused notal bristle polarity defects and those that caused lethality by clonal over-expression using the FLP-out technique. Two independent RNAi lines knocking down each of *cul1* and *skpA* showed wing hair polarity defects including swirling patterns and multiple hairs. This phenotype was also observed upon induction of *cul1* mutant clones (Fig 1A, 1C, and 1E). Pre-hairs within *cul1* and *skpA* knock-down clones showed abnormal polarity, and delayed emergence, as is frequently seen in PCP mutants (Fig 1B, 1D, and 1F) [1]. Furthermore, pre-hairs in neighboring wild-type cells, up to five to six cells away, grew toward mutant clones (Fig 1B, 1D, and 1F), suggesting that the Cul1 complex affects a PCP process that acts both cell autonomously and non-autonomously. Cul1 and SkpA are components of the SCF E3 ligase complex, known to regulate the cell cycle and various signaling pathways including the Wnt and Hedgehog pathways [40,41]. Our screen suggests that they may also work together to regulate hair polarity. F-box protein components of the SCFs sequester substrates to the complex for ubiquitinylation, thereby determining substrate specificity of the E3 complex [42]. Similar to *cul1* and *skpA*, knock-down clones of the F-box protein Slimb produce retarded and abnormally polarized trichome emergence, and showed domineering non-autonomy (Fig 1G), suggesting that Slimb is the F-box protein mediating the function of the Cul1 complex in trichome polarization. Notably, Slimb has recently been implicated in regulating the Par-3/Par-6/aPKC complex to control polarity of the *Drosophila* oocyte, follicle cells, and imaginal disc cells [43,44].

Clones mutant for cell-cycle regulators show multiple hairs that correlate with irregular cell shape and size, and that are reminiscent of those seen in Cul1 complex mutant clones [45,46]. However, these phenotypes are not closely correlated in SCF complex knock-down or mutant clones, such that even clones with relatively normal cell shape and size show profound polarity defects. Furthermore, retardation of hair growth and non-autonomous hair polarity defects were not observed for cell-cycle regulator mutants. Polarity defects caused by Cul1 complex mutation are therefore not likely to result from cell cycle-dependent alterations of cell size and shape.

### The Cul1 complex regulates junctional levels and asymmetric localization of core PCP proteins

**Fig 2:**
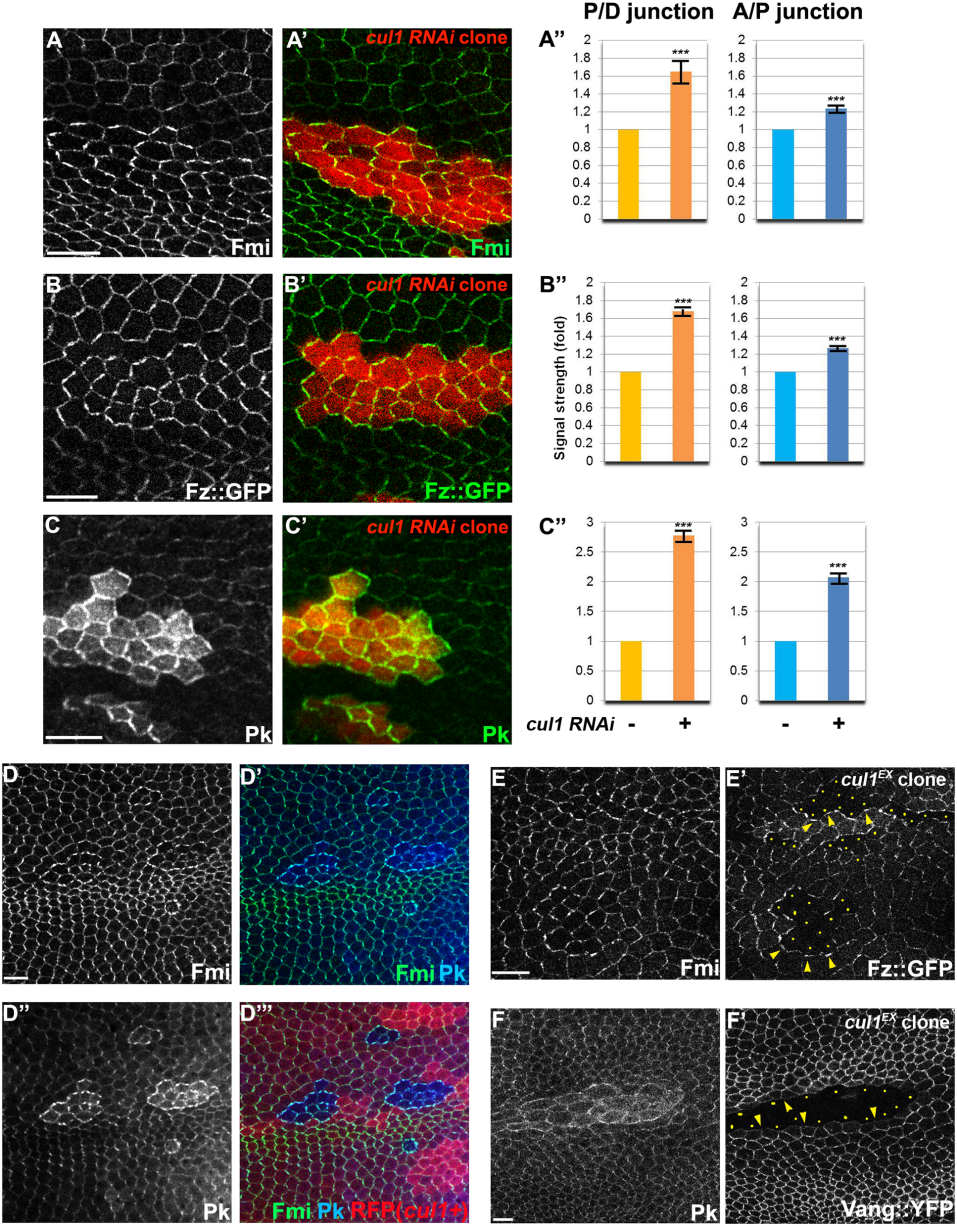
The Cul1 complex regulates accumulation of core PCP components. Apical patterns of core PCP components bearing *cul1* knock-down (RFP positive in A-C,). and mutant (D; homozygous for *cul1* ^*EX*^, RFP negative) clones at 28hr APF. A”, B”, C”; quantification of fold differences for respective core proteins at junctions between *cul1 RNAi* (+) or wildtype (-) cells. ***P<0.0001; t-test. Endogenous Fmi (green in A and D) and Pk (green in C and blue in D) were labeled with antibodies and Fz::GFP,was expressed by heterologous promoters (green in B). All core components tested show enriched apical membrane staining in knock-down or mutant clones compared to surrounding wild-type cells (see also S1 Fig). Core proteins in wild-type cells near clones are misoriented, with Fz::GFP sequestered (E’) and Vang::YFP repelled (F’) in neighboring cells. Absence of Fz::GFP (E) and Vang::YFP (F) signal labels *cul1* mutant (*cul1* ^*EX*^) clones, and arrowheads in E and F point to each in the abutting membrane of neighboring wild-type cells. Yellow dots in E’ and F’ indicate *cul1* mutant cells neighboring wildtype cells. Scale bars: 10μm. Genotypes are (A) *y*, *w*, *hsflp/+; UAS-cul1* ^*IR108558*^ */+; actP>CD2>GAL4*, *UAS-RFP/+*, (B) *y*, *w*, *hsflp/+; UAS-cul1* ^*IR108558*^ */armP-fz*::*GFP; actP>CD2>GAL4*, *UAS-RFP/+*, (C) *y*, *w*, *hsflp/+; UAS-cul1* ^*IR108558*^ */+; actP>CD2>GAL4*, *UAS-RFP/+*, (D) *y*, *w*, *hsflp/+(Y); FRT42D*, *cul1* ^*EX*^ */FRT42D*, *ubiP-NLS*::*mRFP*, (E) *y*, *w*, *hsflp/+(Y); FRT42D*, *cul1* ^*EX*^ */FRT42D*, *armP-fz*::*GFP*, (F) *y*, *w*, *hsflp/+(Y); FRT42D*, *cul1* ^*EX*^ */FRT42D*, *actP-vang*::*YFP*.

The nature of the *cul1* clonal non-autonomy is more similar to that seen with core module components as compared to global module components. We therefore explored whether the Cul1 complex could regulate a core protein(s). Fmi, Fz, Dsh, Vang, and Pk, all accumulated to elevated levels at the apical membrane in Cul1 complex knock-down and mutant clonal wings (Fig 2A–2E and S1), suggesting that the Cul1 complex indeed regulates core proteins.

The domineering non-autonomy associated with Cul1 complex mutant or knock-down clones shows hairs in wild-type cells growing toward the clones. Since trichomes emerge on the side of the cell where the ‘distal’ proteins Fz, Dsh, and Dgo, are located, we examined whether *cul1* mutant cells affect the polarity of neighboring cells by recruiting distal core proteins and repelling proximal core proteins at the adjacent boundary of neighboring cells. Fz::GFP or Vang::YFP expressed only in wild-type cells surrounding *cul1* mutant clones show that *cul1* mutant cells attract Fz::GFP but repel Vang::YFP (Fig 2E and 2F). This phenotype resembles overexpression of proximal core proteins or loss of the distal core protein Fz [30], suggesting that Cul1 complex mutation either up-regulates a proximal or down-regulates a distal core protein(s).

### Cul1-mediated effects on core PCP proteins require Pk

To identify the potential target of Cul1, we compared the profile of effects of *cul1* knock-down or mutant clones on hair polarity and the levels of other core proteins to those caused by either loss-of-function or overexpression of each of the other core components (S1 Table). Like *cul1* knock-down or mutant clones, only Pk overexpression reorients hairs in neighboring cells to point toward the clone and induces elevated levels of all other core factors at intercellular junctions. Furthermore, we and others have observed that, like *cul1* knock-down or mutant clones, Pk over-expression induces co-clustering of Fmi, Fz, and Dsh at membrane domains where Pk accumulates [19,33]. These observations suggest that Pk is a likely target of Cul1 complex mediated degradation. These results do not rule out the possibility that other unknown factor(s) or other core proteins might also be regulated by the Cul1 complex to control PCP.

**Fig 3:**
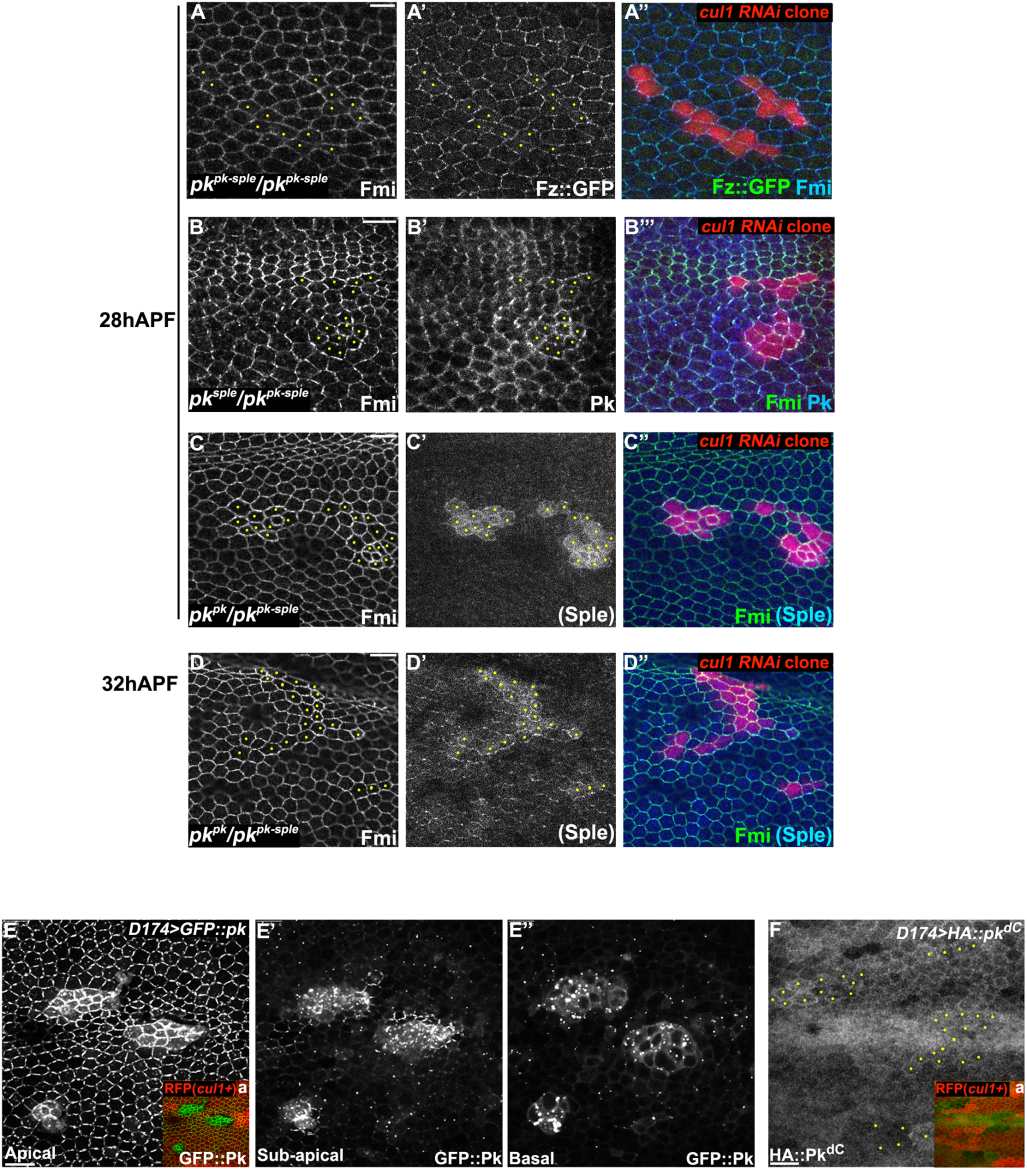
Pk is required for post-transcriptional Cul1 complex-mediated core control. Apical patterns of Fz::GFP (green) and Fmi (blue) in *pk* ^*pk-sple13*^ */pk* ^*pk-sple14*^ pupal wings bearing *cul1* knock-down clones (red, RFP) (A). *cul1* knock-down clones (red, RFP) were also generated in *pk* ^*pk-sple14*^ */pk* ^*sple1*^ (B) and *pk* ^*pk-sple14*^ */pk* ^*pk30*^ (C, D) wings. Fmi (green, B-D), Pk^*Pk*^ (blue, B) and PK^*Sple*^ (blue, C and D) isoforms were labeled by antibody staining (the anti-Pk antibody recognizes both isoforms; in C’ and D’, signal was assumed to reflect Pk^*Sple*^ since the Pk^*Pk*^ isoform is not expressed). The presence of either single isoform enables apical core protein enrichment (compare A with B, C, and D) in *cul1* knock-down clones. PK^*Sple*^ staining is very weak, but slightly stronger in at 32 h APF compared to 28 h APF (compare C’ with D’). Orientation of PK^*Sple*^ staining corresponds to expected hair direction in *pk* ^*pk*^ */pk* ^*pk-sple*^ mutant wings. *D174GAL4* driven GFP::Pk (E), but not HA::pk^*dC*^ (F), accumulates in *cul1* mutant (*cul1* ^*EX*^) clones (mutant clones without RFP indicated in Ea and Fa) in 28hr APF wings. In A-D and F, yellow dots are indicating *cul1* knock-down or mutant cells. Scale bars: 10μm. Genotypes are (A) *y*, *w*, *hsflp/+; UAS-cul1* ^*IR108558*^, *pk* ^*pk-sple14*^ */pk* ^*pk-sple13*^ *; actP>CD2>GAL4*, *UAS-RFP/armP-fz*::*EGFP*, (B) *y*, *w*, *hsflp/+; UAS-cul1* ^*IR108558*^, *pk* ^*pk-sple14*^ */pk* ^*sple1*^ *; actP>CD2>GAL4*, *UAS-RFP/+*, (C, D) *y*, *w*, *hsflp/+; UAS-cul1* ^*IR108558*^, *pk* ^*pk-sple14*^ */pk* ^*pk30*^ *; actP>CD2>GAL4*, *UAS-RFP/+*, (E) *y*, *w*, *hsflp/D174GAL4; FRT42D*, *cul1* ^*EX*^ */FRT42D*, *ubiP-NLS*::*mRFP; UAS-GFP*::*pk/+*, (F) *y*, *w*, *hsflp/D174GAL4; FRT42D*, *cul1* ^*EX*^ */FRT42D*, *ubiP-NLS*::*mRFP; UAS-HA*::*pk* ^*dC*^ */+*.

If Pk is a principle target of Cul1 in PCP signaling, the effects of *cul1* knock-down or mutation should be suppressed by loss of Pk. To test this, *cul1* RNAi clones were generated in *pk* ^*pk-sple*^ mutant wings and patterns of core proteins were analyzed (Fig 3A). Effects of *cul1* RNAi on core protein accumulation were abolished in *pk* ^*pk-sple*^ mutant wings, such that, for both Fmi and Fz, signal strength inside and outside of clones was indistinguishable (Fig 3A). As expected, asymmetry of Fmi and Fz was disrupted in *pk* ^*pk-sple*^ wings. Pk is therefore required for the Cul1 complex to act on the core mechanism, and taken together with the full phenocopy of *cul1* knock-down or mutation by Pk overexpression (sufficiency; S1 Table), the complete blockade (necessity) suggests that no other Cul1 targets play a significant role in Cul1’s ability to modify core PCP activity.

*Drosophila* is believed to have three Pk isoforms, two of which, Pk^Pk^ and Pk^Sple^, are important for epithelial planar cell polarity [12]. Hair polarity in the wing requires Pk^Pk^ but not Pk^Sple^, although some Pk^Sple^ is expressed [27]. We therefore examined whether both isoforms are regulated by the Cul1 complex. Fmi protein accumulated in *cul1* RNAi clones generated in either a *pk* ^*pk*^/*pk* ^*pk-sple*^ or *pk* ^*sple*^/*pk* ^*pk-sple*^ background (Fig 3B–3D), indicating that Cul1 regulates both the Pk^Pk^ and Pk^Sple^ isoforms. Though our antibody detects only very low levels of diffuse Sple signal in *pk* ^*pk*^/*pk* ^*pk-sple*^ wings (signal is assumed to represent Sple since the Pk^pk^ isoform is not expected to be expressed; Fig 3C’ and 3D’), the signal is stronger in *cul1* RNAi clones than in surrounding tissue. Nonetheless, total Pk^Sple^ levels remain well below levels of the Pk^pk^ isoform.

We next asked if Cul1 complex activity acts post-transcriptionally on Pk. Like endogenous Pk, levels of GFP::Pk expressed from a heterologous promoter are increased in 28hr APF *cul1* mutant clones (Fig 3E), thus demonstrating a post-transcriptional regulation of Pk protein levels. The same was true during third instar (S2B and S2C Fig) and 24hr APF pupal wings (S2D and S2E Fig), suggesting that Cul1 is likely required throughout the course of PCP signaling. These data are consistent with Pk being a ubiquitinylation substrate of the Cul1 complex, though they do not rule out an indirect effect.

### Pk is a target for Cul1 complex-mediated ubiquitinylation

**Fig 4:**
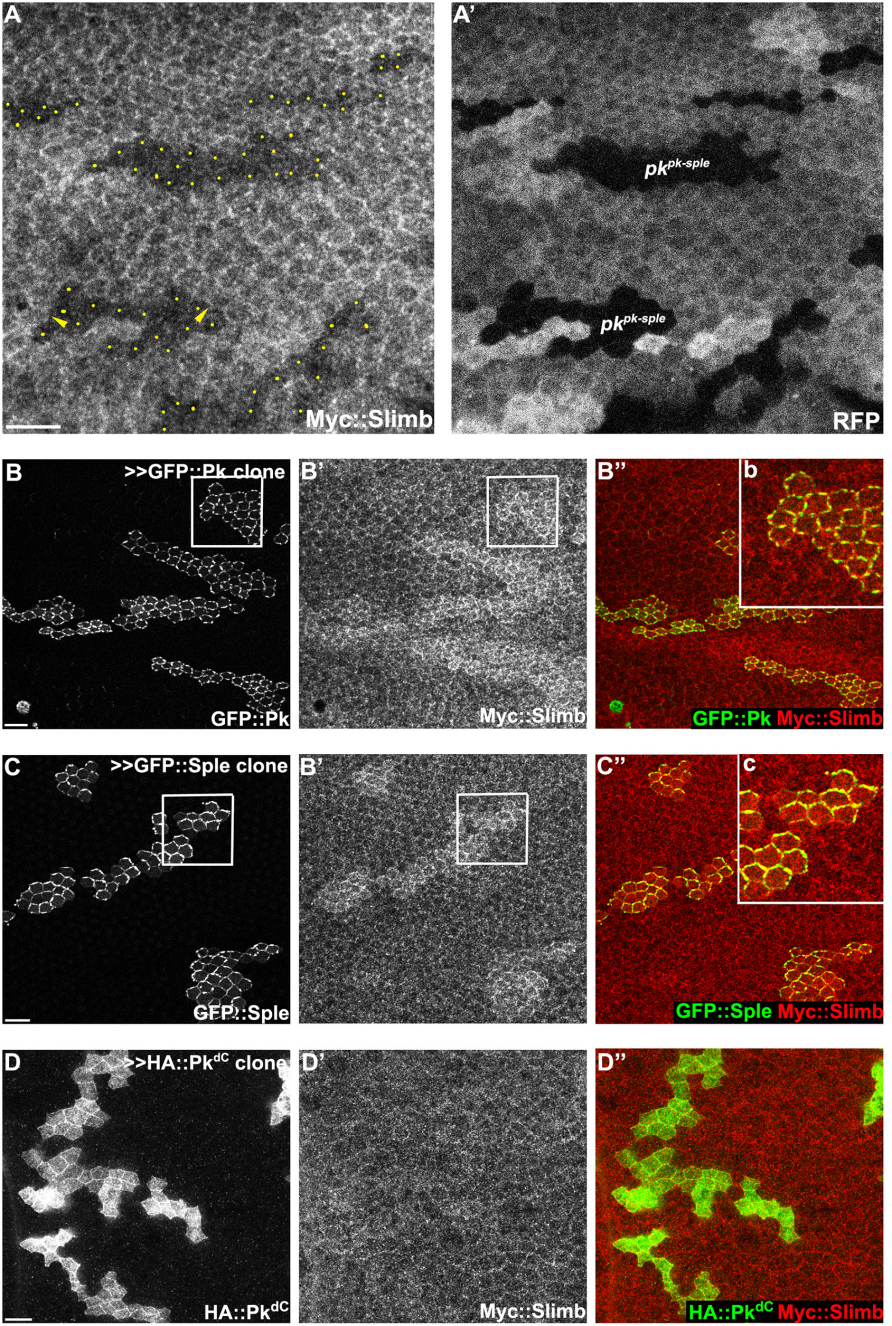
Slimb interacts with Pk and accumulation of Slimb depends on *pk*. (A) Apical Myc::Slimb staining is diminished in *pk* ^*pk-sple13*^ homozygous mutant clones (absence of RFP in A’). Yellow dots in A indicate mutant cells at the clonal border (A’). Asymmetric localization of Myc:Slimb is subtle, but can be appreciated by differential enrichment at boundaries of *pk* ^*pk-sple13*^ clones (arrowheads; see also S3 Fig). Clonal overexpression of GFP::Pk (green, B) and GFP::Sple (green, C), but not HA::Pk^*dC*^ (green, D), sequesters Myc::Slimb (red); *tubP-6XMyc*::*slimb* was detected with anti-c-Myc antibodies (see S3C Fig for demonstration of antibody specificity). (B”b and C”c) magnified images of areas indicated with white squares in B and C. All samples are prepared from 26-28h APF pupae. Scale bars: 10μm. Genotypes are (A) *y*, *w*, *hsflp/+(Y); FRT42D*, *pk* ^*pk-sple13*^ */FRT42D*, *ubiP-NLS*::*mRFP; tubP-6XMyc*::*slimb/+*, (B) *y*, *w*, *hsflp/+; tubP-6XMyc*::*slimb/+; actP>CD2>GAL4*, *UAS-RFP/UAS-GFP*::*pk*, (C) *y*, *w*, *hsflp/+; tubP-6XMyc*::*slimb/+; actP>CD2>GAL4*, *UAS-RFP/UAS-GFP*::*sple*, (D) *y*, *w*, *hsflp/+; tubP-6XMyc*::*slimb/+; actP>CD2>GAL4*, *UAS-RFP/ UAS-HA*::*pk* ^*dC*^.

To test whether Pk could be a target of the Cul1 complex, we sought evidence of a physical interaction between Pk and the Cul1 complex. As Slimb appeared to be the F-box protein providing substrate specificity to the Cul1 complex for its PCP function, we first asked whether Slimb might colocalize with Pk. Consistent with this possibility, in wildtype tissue, ubiquitously expressed Myc::Slimb protein was detected at the apical cell boundary, with a very subtle asymmetric localization that appears to be on the proximal side of the cell (arrow heads in Figs 4A and S3A–S3C). In *pk* ^*pk-sple*^ mutant clones, Myc::Slimb staining was diminished both at the apical cell junctions and also more basally in the absence of Pk (Fig 4A), indicating that retention of Slimb protein depends on Pk. Furthermore, in wildtype tissue near a clone that induces domineering non-autonomy, orientations of Pk and Myc::Slimb are coordinately reorganized (S3C Fig).

To further assess dependence of Slimb localization on Pk, GFP tagged Pk^Pk^ or Pk^Sple^ isoforms were clonally overexpressed in wings ubiquitously expressing 6XMyc::Slimb. Upon GFP::Pk or GFP::Sple overexpression, Myc::Slimb was sequestered at the apical membrane (Fig 4B and 4C), suggesting that they might physically form a complex. In contrast, clonal overexpression of Fmi::YFP failed to sequester Myc::Slimb (S3A Fig). Since Fmi overexpression causes Fz and Dsh accumulation [47], neither Fmi, Fz or Dsh cause Slimb accumulation. Vang overexpressing clones showed slightly higher Myc::Slimb staining (S3B Fig), though less robust than Pk^Pk^ or Pk^Sple^ overexpression.

Previous studies showed that F-box proteins are often ubiquitinylated by their own Cul complex in the absence of other substrates, and this provides a mechanism by which they can exchange their F-box proteins and substrates [48,49]. Consistent with this, Myc::Slimb protein levels were increased in *cul1* knock-down clones (S3D Fig). More importantly, these results support the possibility that both Pk and Sple isoforms are substrates of the Cul1 complex.

**Fig 5:**
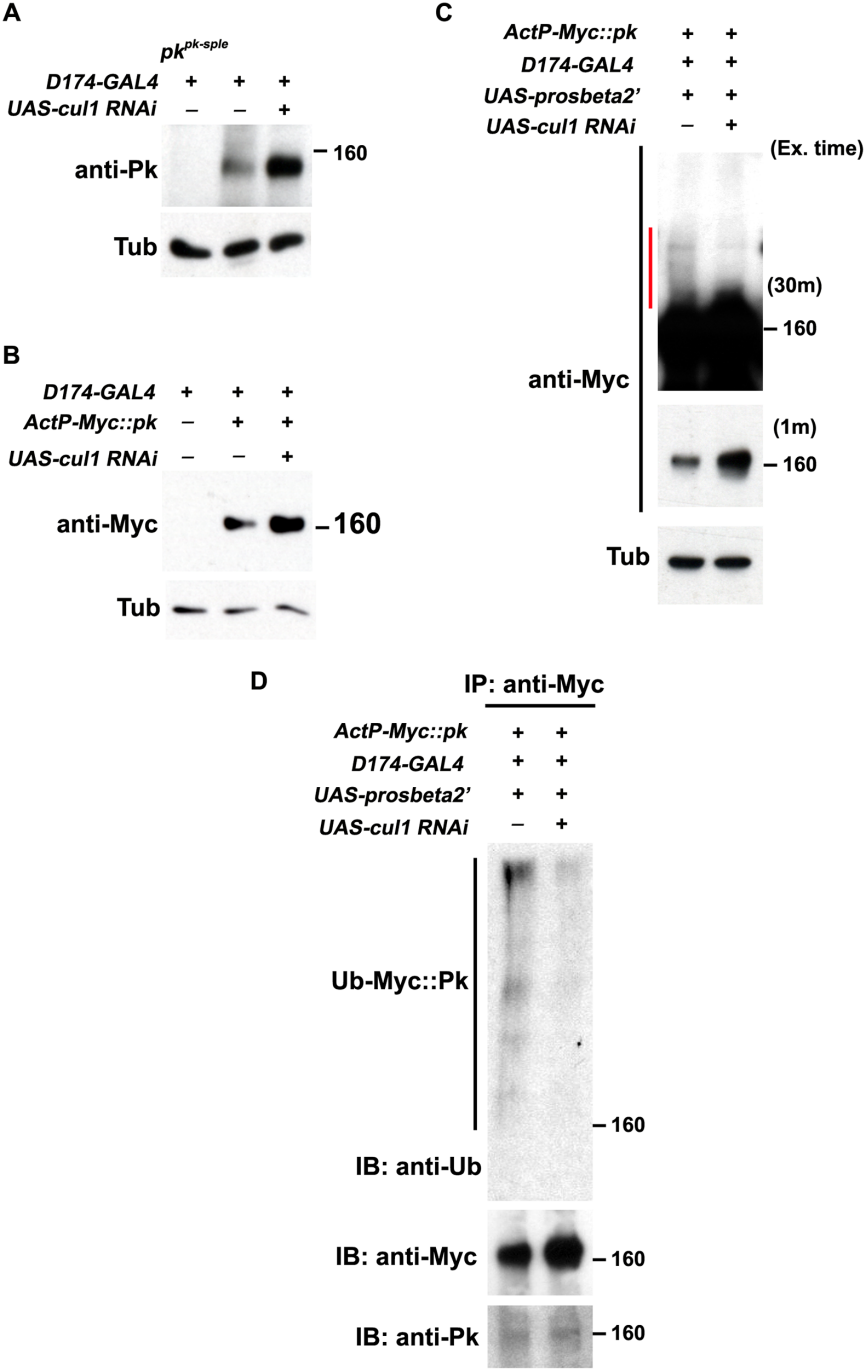
Pk protein level is regulated by Cul1-mediated ubiquitinylation and proteasomal degradation. Levels of endogenous Pk (A) and Myc::Pk expressed with the actin promoter (B) were examined by western blot. Knocking-down *cul1* using a *D174GAL4* driven *cul1* RNAi construct increases the amount of Pk or Myc::Pk in third instar wing discs (lane 3 in A and B). Overexpression of *UAS-prosbeta2’* induces modification of Myc::Pk (labeled by a red line in C) that is *cul1* dependent (C; compare lane1 with lane 2). Note that the protein level of unmodified form of Myc::Pk is higher with *cul1i* in the blot with short exposure time. γ-Tubulin was probed as loading controls (A-C). (D) Myc::Pk from wing discs with genotypes as in (C) was immunoprecipitated and detected with anti-Ub, anti-Myc and anti-Pk antibodies. More Ub signal was detected in the absence as compared to the presence of *cul1* knock-down. Genotypes are (A) Lane 1: *D174GAL4/+(Y); pk* ^*pk-sple13*^ */ pk* ^*pk-sple14*^, Lane 2: *D174GAL4/+(Y)*, Lane 3: *D174GAL4/+(Y); UAS-cul1* ^*IR108558*^ */+*, (B) Lane 1: *D174GAL4/+(Y)*, Lane 2: *D174GAL4/+(Y); +/+; actP-6XMyc*::*pk/+*, Lane 3: *D174GAL4/+(Y); UAS-cul1* ^*IR108558*^ */+; actP-6XMyc*::*pk/+*, (C) Lane 1: *D174GAL4/UAS-lacZi; +/+; actP-6XMyc*::*pk/UAS-prosbeta2’*; Lane 2: *D174GAL4/+(Y); UAS-cul1* ^*IR108558*^ */+; actP-6XMyc*::*pk/UAS-prosbeta2’*, (D) same as (C).

If Pk is a substrate for the Cul1 complex, and if ubiquitinylation targets it for degradation, then in addition to co-dependent localization, levels of Pk should be dependent on Cul1. The elevation of endogenous Pk or exogenous Myc::Pk levels upon *cul1* knock-down as shown by immunofluorescence was corroborated by increased levels detected by Western blot in *cul1* knock-down wing discs (Fig 5A and 5B), consistent with Cul1 dependent destabilization of Pk protein.

Furthermore, If Pk is a substrate for Cul1 complex activity that targets for proteasomal degradation, then uibquitinylated Pk should be detected when proteasome activity is impaired. We identified conditions in which expression of the dominant negative form of the proteasomal subunit, Prosbeta2’ knocked down proteasome activity without causing lethality. Under these conditions, we detected slowly migrating forms of Myc::Pk, consistent with ubiquitinylation, that were diminished upon *cul1* knock-down, suggesting that these are ubiquitinylated forms of Pk (Fig 5C). Finally, we verified that the slowly migrating forms of Pk are indeed ubiquitinylated by immunoprecipitating Myc::Pk from wing discs with knocked down proteasome activity, either with or without *cul1* knock-down, and probed for Ubiquitin (Fig 5D). Ubiquitin signal was stronger without *cul1* knock-down; therefore, levels of ubiquitinylated Pk depend on Cul1 activity.

These results together argue that elevated endogenous Pk or exogenous Myc::Pk levels in *cul1* knock-down wing discs (Fig 5A and 5B) are caused by defects in Cul1-mediated ubiquitinylation and proteasomal degradation of Pk. We do not rule out the unlikely possibility that the Cul1 complex, acting in close proximity to Pk, acts on an intermediate substrate that in turn regulates ubiquitinylation of Pk.

Overexpression of a C-terminal deleted form of Pk^Pk^ (HA-Pk^dC^; aa1-472; supplemental information) showed less membrane localization and greater expression in the cytosol and nucleus as compared to wild type Pk^Pk^, as expected due to deletion of both the CaaX sequence and Vang binding domains (S4E and S4F Fig), This truncated Pk did not accumulate in *cul1* mutant clones (Fig 3F) nor did it sequester Myc::Slimb (Fig 4D), suggesting the possibility that the C-terminal domain of Pk contains one or more Slimb binding sites, though these observations may also be explained by reduced membrane recruitment or failure to interact with Vang. Of note, USPX9, the human de-ubiquitinylating ortholog of Fat Facets (Faf), interacts with the C-terminus of vertebrate Pk1 and Pk2, suggesting C-terminal ubiquitinylation of these proteins [50]. Additional work will be required to more precisely define the regions of Pk required for Cul1 ubiquitinylation

### Pk requires Vang to accumulate and cluster core PCP proteins

Levels of each of the core PCP proteins must be regulated to achieve normal polarization. Our results thus far indicate that the Cul1 complex regulates Pk levels. Previous studies also showed that farnesylation and association with Vang are required to regulate Pk levels [33,38]. However, the function of Pk in core PCP function is not well understood, and these results fail to illuminate how excess Pk perturbs normal core PCP function.

Core PCP proteins assemble into asymmetric complexes, with Fmi homodimers spanning intercellular junctions, and the proximal proteins Vang and Pk associated with one side, and the distal proteins Fz, Dsh and Dgo associated with the other side of the complex ([Dgo-Dsh-Fz-Fmi]-[Fmi-Vang-Pk]). Asymmetric subcellular localization represents the preferential accumulation of this complex oriented in one direction and exclusion of the oppositely oriented complex. Pk has been suggested to mediate a mutual exclusion to facilitate this function via feedback [19]. Pk has also been implicated in stabilizing [Fz-Fmi]-[Fmi-Vang] complexes [19,33,47].

**Fig 6:**
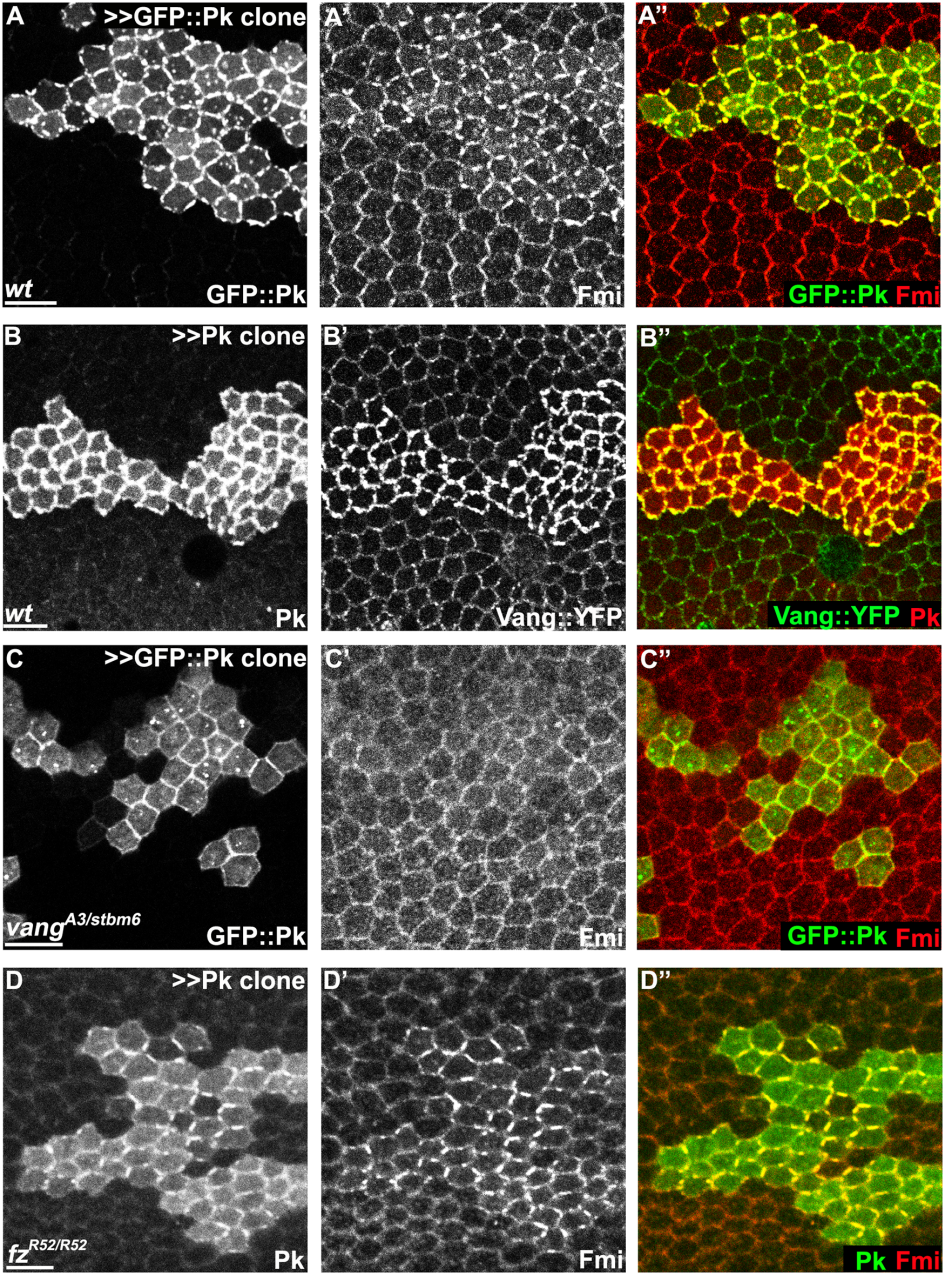
Pk-mediated apical clustering of core proteins requires Vang. Apical clustering of Fmi (red) is seen in cells clonally overexpressing GFP::Pk (green, A) and Pk (green, D) in wild-type (A) and *fz* mutant (*fz* ^*R52*^ */fz* ^*R52*^, D) but not in *vang* mutant (*vang* ^*A3*^ */vang* ^*stbm6*^, C) wing tissues (26hr APF). Apical clustering of Vang::YFP (green, B) with overexpression of Pk (red, B) is shown. Majority of GFP::Pk, or all of Pk, positive puncta are also positive for Fmi, or Vang::YFP, respectively (A, B). Scale bars: 10μm. Genotypes are (A) *y*, *w*, *hsflp/+; +/+; actP>CD2>GAL4*, *UAS-RFP/UAS-GFP*::*pk*, (B) *y*, *w*, *hsflp/+; UAS-pk/; actP>CD2>GAL4*, *UAS-RFP/actP-vang*::*YFP*, (C) *y*, *w*, *hsflp/+; vang* ^*A3*^ */vang* ^*stbm6*^ *; actP>CD2>GAL4*, *UAS-RFP/UAS-GFP*::*pk*, (D) *y*, *w*, *hsflp/+; UAS-pk/actP>CD2>GAL4*, *UAS-GFP; fz* ^*R52*^ */fz* ^*R52*^.

Though the mechanism by which Pk carries out its function is unknown, it has been known for some time that overexpression of Pk induces co-clustering of core proteins Fmi, Dsh and Vang at junctional domains [19,33]. Clustering of Fmi does not require Fz [33]. As Pk interacts with Vang, it has been assumed that Pk acts via Vang to stabilize intercellular bridges. Consistent with this idea, we observed that upon Pk overexpression, co-clustering of Pk and Fmi does not depend on Fz, but does indeed depend on Vang (Fig 6A–6D). Similarly, Pk co-clustering with Fmi at apical junctions in *cul1* knock-down clones is observed in *fz* mutant wings (S2A Fig). These results confirm that Pk can induce clustering of Fmi complexes through Vang, but independent of the distal core protein, Fz ([Fmi]-[Fmi-Vang-Pk]). We note that while Pk overexpression can induce accumulation of Vang in the absence of Fmi, the characteristic clustering is not observed (S4A and S4B Fig).

### Pk mediates Vang-Fmi internalization and routing to intracellular vesicular compartments

**Fig 7:**
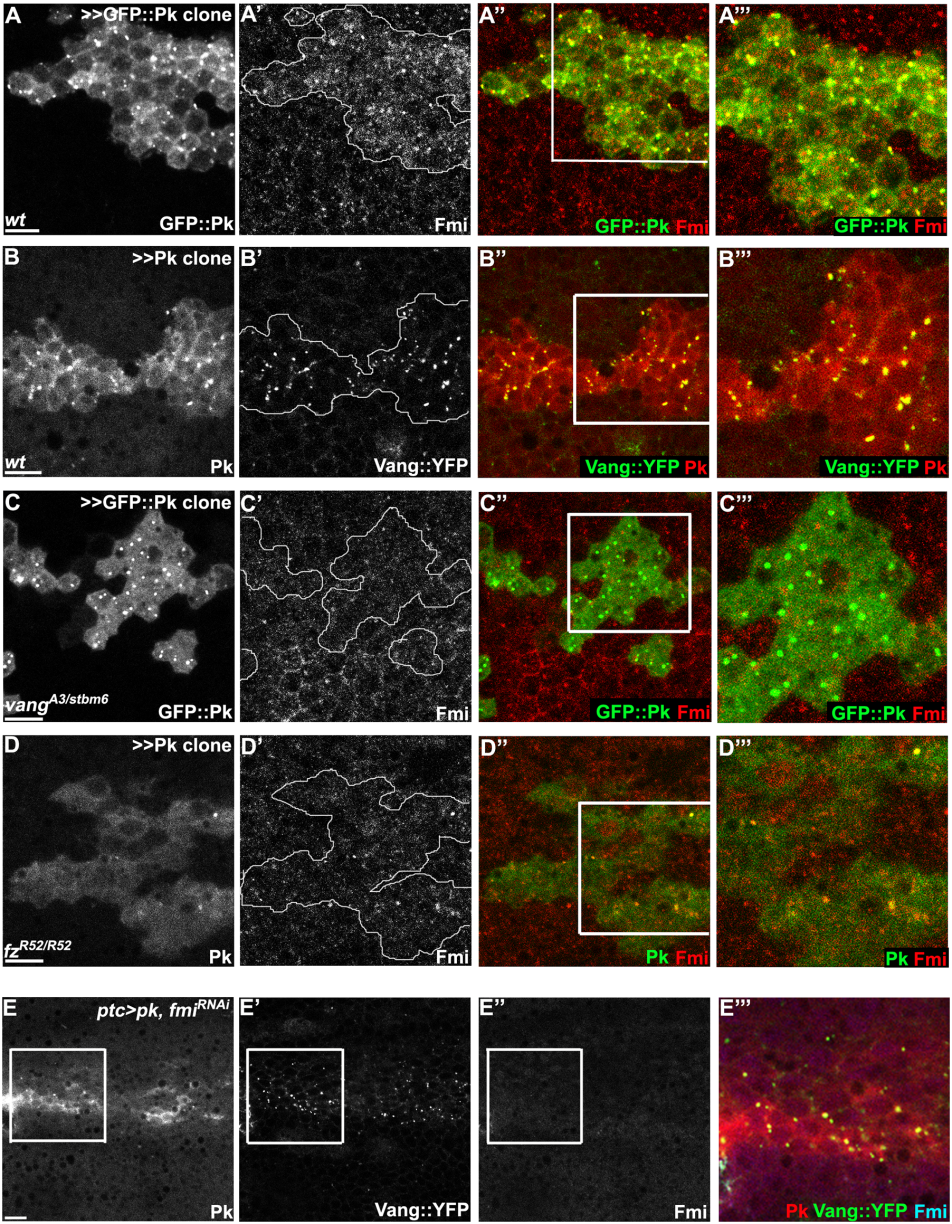
Vang-Fmi internalization by Pk depends on Vang and Fz. (A-D) Sub-apical puncta positive for Pk, Fmi, or Vang::YFP in the same clones shown in Fig 6A–6D. Overexpression of *GFP*::*pk*, or *pk*, induces Fmi, or Vang::YFP, positive puncta (A, B). Most puncta positive for Fmi (red, A) are also positive for GFP::Pk and all Pk positive puncta are also Vang::YFP positive (B) Fmi-positive puncta were not induced or co-labelled with GFP::Pk-positive puncta in the absence of *vang* (C). In the absence of *fz*, GFP::Pk-positive puncta are less frequent, yet those seen co-label with Fmi (D). Without *fz*, Fmi-positive puncta are less frequent or less bright with or without *pk* overexpression (see Fmi staining in- and outside of *pk* overexpressing clones in D and compare with A). (E) Vang::YFP puncta are induced by *ptc-GAL4* driven *pk* overexpression when *fmi* is simultaneously knocked down, showing that Fmi is not absolutely required for Pk dependent Vang puncta formation (Pk, red; Vang::YFP, green; Fmi, blue). Clones are outlined and magnified areas are indicated by white squares. Scale bars: 10μm. Genotypes for A-D are same as those in Fig 6. Genotype for E is, *UAS-Dcr2/+; ptc-GAL4/UAS-pk; actP-vang*::*YFP/UAS-fmi* ^*IRFBst0026022*^.

While Pk overexpression induces clustering and enrichment of core components at apical junctions, several observations suggested additional functions. First, in addition to clustering at the apical cell membrane, we noticed that overexpression greatly increased the amount of GFP::Pk that was localized in cytosolic puncta, presumably consisting of vesicles, that are observed from apical to basal regions of the cell (apical planes in Fig 6A and 6B, and 6C, sub-apical planes in Fig 7). Furthermore, formation of Fmi vesicles was also induced, and Fmi staining co-localized with GFP::Pk (most Fmi positive puncta were also GFP::Pk positive) (Figs 6A and 7A). We infer that these double labeled puncta are also positive for Vang since nearly all Pk positive puncta in Pk overexpressing cells are also positive for Vang::YFP (Figs 6B and 7B).

**Fig 8:**
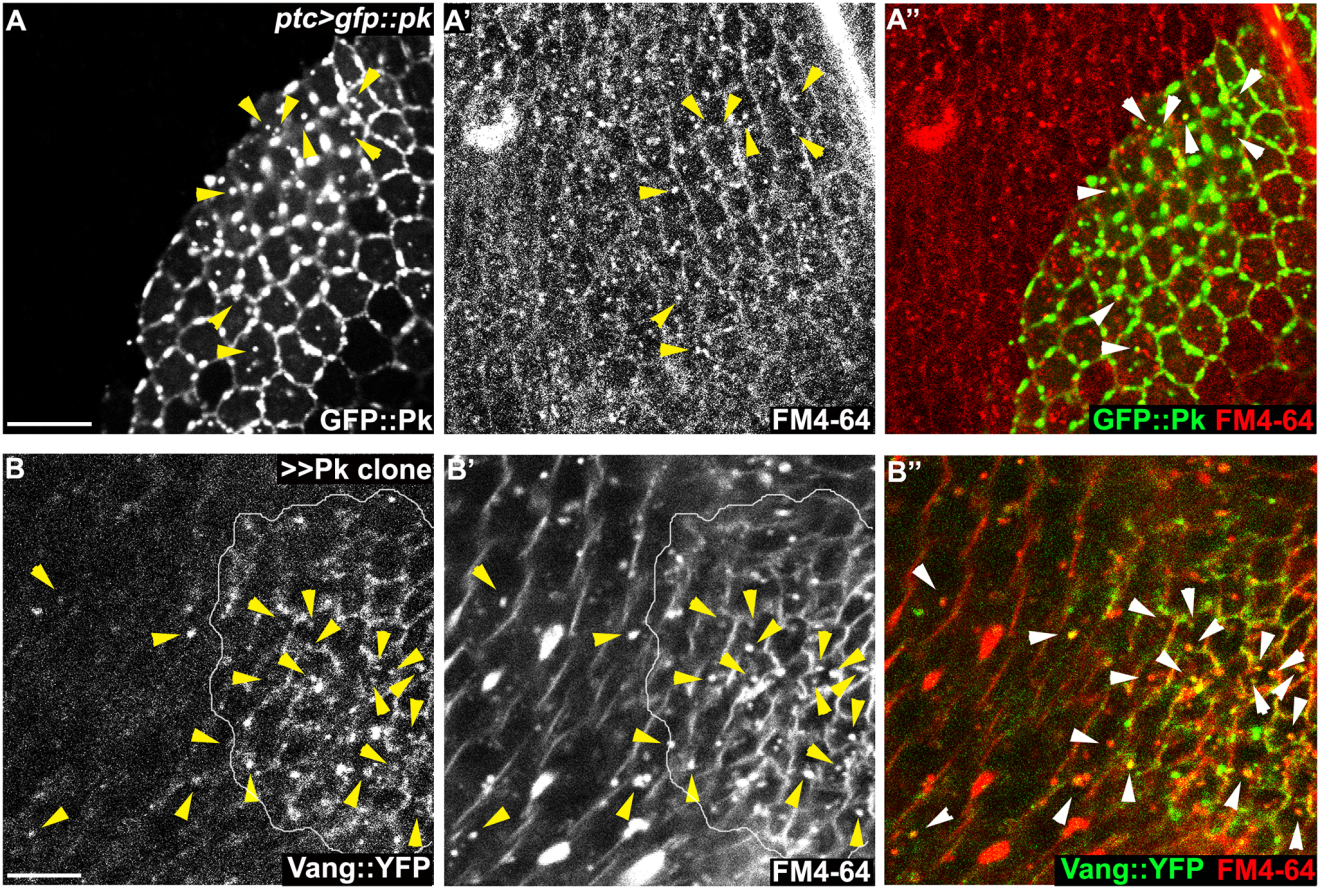
Endocytosis of membranous Pk and Vang. Windows were created over the wing of live 24-26h APF pupae by opening the pupal case and cuticle, and then incubated in FM4-64 solution. Apical or sub-apical puncta for GFP::Pk (green in A) and Vang::YFP (green in B) were then visualized. (A) Many GFP::Pk positive cytosolic puncta (GFP::Pk driven by *ptc-GAL4*) co-labeled with FM4-64. (B) In *pk* overexpressing clones (outlined), Vang::YFP frequently co-labeled with FM4-64. Double positive puncta for GFP::Pk, or Vang::YFP, and FM4-64 are indicated with arrowheads (see the manuscript for quantification). Scale bars: 10μm. Genotypes are (A) *ptc-GAL4/+; UAS-gfp*::*pk*, (B) *actP>CD2>GAL4/y*, *w*, *hsflp; UAS-pk/+; actP-vang*::*YFP*.

To determine whether the Pk dependent puncta are vesicles resulting from an endocytic process, FM4-64 dye uptake assays were carried out. As FM4-64 cannot penetrate the plasma membrane of live cells, FM4-64 positive puncta indicate endocytic vesicles from internalization of plasma membrane [51]. Live pupae with exposed wings were briefly bathed in FM4-64 and live wings directly subjected to confocal microscopy. To assay for internalization of apical [Fmi-Vang-Pk] complexes, apical and subapical puncta were analyzed. Of GFP::Pk (driven by *ptc-GAL4*) positive puncta, 36 to 53% (five wings; Fig 8A) were positive for FM4-64. Similarly, when Pk was clonally overexpressed in the presence of Vang::YFP 55 to 74% of Vang::YFP positive puncta were positive for FM4-64 (Fig 8B;five wings tested). This fraction was increased compared to regions not overexpressing Pk (22 to 41%; five wings tested). Note that these percentages are expected to be lower bounds of the true values due to brief exposure to the dye. These results support the idea that Pk mediates endocytosis of Vang. We then asked whether endogenous levels of Vang vesicles depend on endogenous Pk. Consistent with our conclusion, in the absence of Pk (*pk* ^*pk-sple*^ mutant clones), the number of Vang::YFP positive puncta was reduced to 77% of that in wildtype cells (77± 9%, p = 0.0025; Vang::YFP puncta counted from 412 mutant and 1000 wildtype cells in *pk* ^*pk-sple*^ mutant clones in five wings). Taken together, these results demonstrate that Vang (together with Fmi) is endocytosed with Pk, and that increased Pk stimulates this internalization. Not surprisingly, these data also indicate that a fraction of Vang::YFP positive vesicles are independent of Pk, perhaps representing distinct recycling and/or biosynthetic pathways.

In the absence of Vang, GFP::Pk overexpression still produced GFP::Pk positive vesicles, but Fmi positive internalization was greatly reduced (Figs 6C and 7C), These results are consistent with the model that Pk dependent internalization of Fmi requires Vang. Conversely, depletion of Fmi does not affect Pk dependent internalization of Vang (Figs 7E and S4B for *fmi* knock-down efficiency). Based on these results, we hypothesize that Pk overexpression induces the coordinate internalization of Vang and Fmi through an endocytic pathway, and that internalization of Fmi requires interaction of Pk with Vang. Unlike internalized vesicles containing Fz and Dsh, a substantial fraction of which remain in the apical part of the cell and is seen to transcytose in a microtubule-dependent fashion [26–28], these vesicles do not transcytose, and are likely either recycled or targeted for degradation. While we have examined these vesicles in the context of manipulating Pk levels directly, we also see excess vesicle formation in *cul1* ^*EX*^ mutant clones (S5A Fig), reinforcing the role of Cul1 dependent ubiquitinylation in regulating this function.

An additional observation is consistent with the hypothesis that Pk promotes internalization of Vang and Fmi. While both Pk overexpression and Cul1 depletion lead to increased, clustered core PCP protein distribution, simultaneous Pk overexpression and Cul1 depletion produced even higher levels of Pk, resulting in even greater vesicle formation and depletion of Fmi from apical junctions (S5B, S5C, and S5D Fig). Thus, while moderately elevated Pk causes clustering and accumulation of core components, very high levels result in removal of core components from the membrane. This is consistent with the possibility that rather than being independent, clustering is associated with internalization, with modest levels of clustering and internalization facilitating amplification and extreme levels resulting in depletion of Vang-Fmi from junctions.

### Amplification of asymmetry occurs in clusters

**Fig 9:**
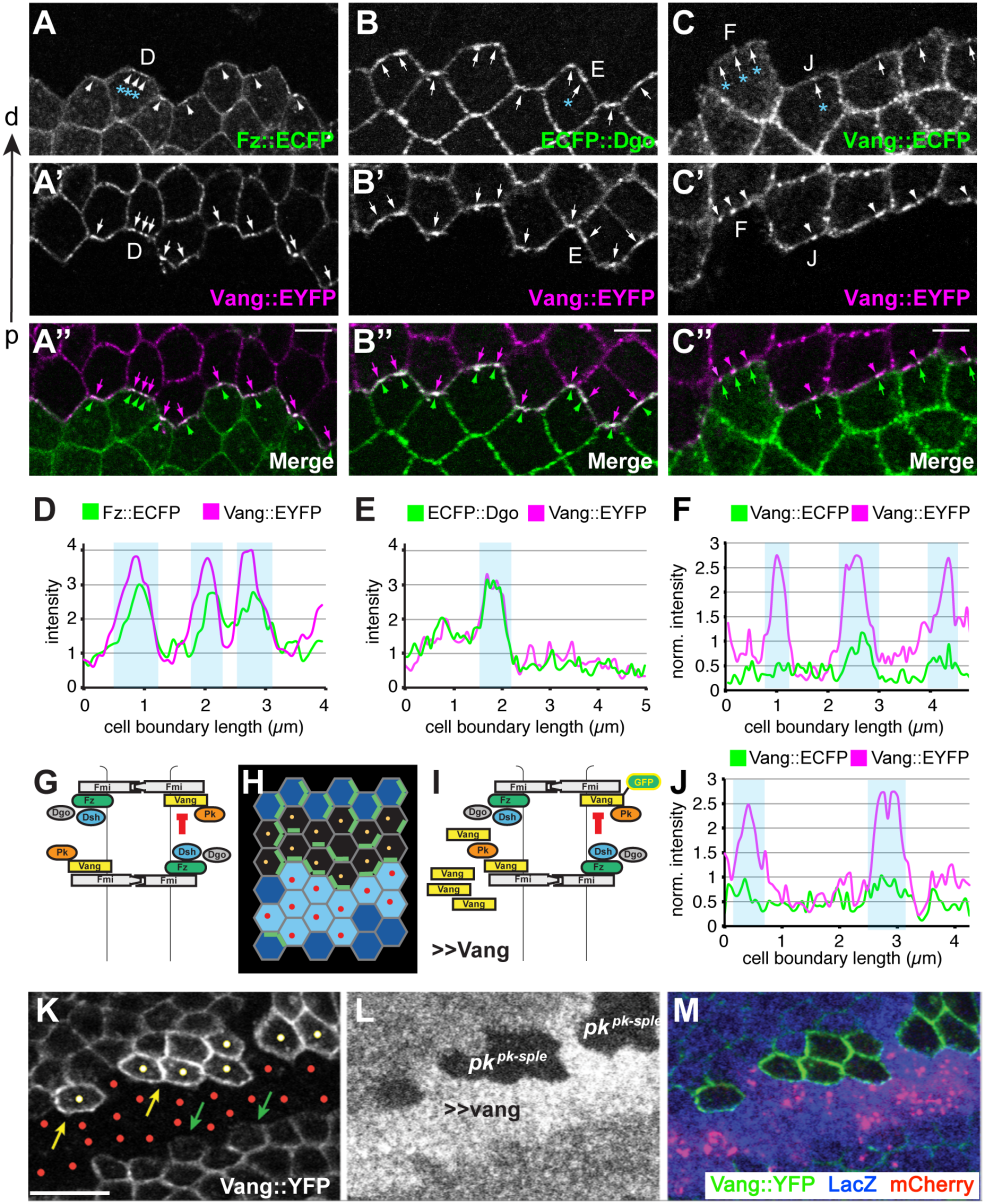
Amplification of asymmetry in membrane clusters and requirement of Pk for excluding Vang. (A-B”) Clone interface between twin spots expressing either a distal PCP protein fused to ECFP (Fz in A, Dgo in B) or Stbm::EYFP (A’,B’). Note that proximal and distal PCP proteins colocalize in puncta at the clone interface, suggesting that puncta connect PCP complexes of adjacent cells with each other. (C-C”) Clone interface between twin spots expressing either Stbm::ECFP or Stbm::EYFP. (D, E) Quantification of intensity (arbitrary units) along the cell boundaries indicated in A and B, and (F, J) quantification of normalized intensity in C. For F and J, intensity levels of cell boundaries along the clone interface were normalized to the average boundary intensity within the respective twin spot. Clones overexpressing Vang were created adjacent to both wild-type and *pk* mutant cells by reverse MARCM (see Supporting Information for genotype) (G-I, K-M). (G) Cartoon showing Fz dependent exclusion of Vang-Fmi complexes. (H) Schematic of reverse MARCM clones; red dots indicate *vang* overexpressing cells (in K-M, red mCherry expressing cells) and yellow dots indicate *pk* mutant clonal cells facing *vang* overespressing cells (H, K). (I) Schematic of MARCM experiment. (K) Vang overexpression excludes Vang::YFP from the membrane in neighboring wild-type cells (green arrows), but fails to exclude Vang::YFP in *pk* mutant cells (yellow dots; yellow arrows indicate membranous Vang::YFP facing *vang* overexpressing cells in *pk* mutant cells). (A-C) 16hr APF and (K-M) 26hr APF wing tissues. Scale bars: 5 μm for A-C; 10μm for K-M. Genotypes are (A) *y*, *w*, *hsflp/+(Y);; ubiP-fz*::*ECFP*, *FRT80 / ubiP-vang*::*EYFP*, *FRT80*, (B) *y*, *w*, *hsflp/+(Y);; ubiP-ECFP*::*dgo*, *FRT80 / ubiP-vang*::*EYFP*, *FRT80*, (C) *y*, *w*, *hsflp/+(Y);; ubiP-vang*::*ECFP*, *FRT80 / ubiP-vang*::*EYFP*, *FRT80*, (K-M) *y*, *w*, *hsflp/D174GAL4; FRT42D*, *armP-LacZ /FRT42D*, *pk* ^*pk-sple13*^, *actP-vang*::*YFP*, *tubP-GAL80; UAS-mCherry/UAS-vang*.

In contrast to clustering, Pk-dependent Fmi vesicle formation was substantially diminished in *fz* mutant tissue (Figs 6D and 7D). The requirement of Fz for Pk to stimulate Fmi-Vang-Pk vesicle formation suggests that it is the interaction between full length complexes, most likely within clusters of complexes in opposite orientations that promotes internalization (Fig 9G). This conclusion is further supported by the observation that clones overexpressing Pk, which induce recruitment of Fz to the neighboring cell boundaries and therefore hairs to point toward the clone, simultaneously induce Vang vesicle formation in the adjacent cells (S6 Fig). We note that in *fz* mutant clones, apicolateral Fmi, the pool from which vesicles may be drawn, is modestly reduced, perhaps by a factor of two [47]. However, there is a greater than 10 fold reduction in vesicle formation, suggesting reduced apicolateral Fmi alone cannot explain the decrease in vesicles. The same is true for *vang* clones.

Fz-dependent internalization of Fmi-Vang-Pk vesicles is likely to be involved in the generation of asymmetry, as competition between oppositely oriented complexes leading to dose-dependent removal of the less abundant complex would amplify asymmetry. Additional evidence for the idea that clustering might be associated with internalization and amplification comes from visualization of asymmetric localization. Asymmetric localization can be visualized by generating twin clones expressing PCP proteins tagged with different fluorophores, and examining shared cell boundaries. This experiment was performed at 16 h APF, when asymmetric localization is detectable, but incomplete, as assessed by traditional methods. As expected, labeling one clone with a distal protein, Fz::ECFP or ECFP::Dgo, and the adjacent clone with the proximal protein Vang::EYFP, shows overlapping accumulation of distal and proximal proteins, with some clustering in puncta (Fig 9A, 9B, 9D, and 9E). In contrast, when both twin clones are labeled with the same protein, in this case Vang::ECFP and Vang::EYFP, asymmetry would be reflected by unequal amounts on the distal and proximal sides of the boundary. Notably, we observed unequal amounts of proximal and distal Vang in puncta, but not in the more diffusely localized protein (Fig 9C, 9F, and 9J). This observation indicates that the generation of asymmetry in core PCP protein complexes is occurring within clusters.

### Pk is required for membrane exclusion of Vang-Fmi complexes

Pk is not needed for assembly of complexes including [Fz-Fmi]-[Fmi-Vang], but does play a role in stabilizing these complexes, perhaps via clustering [32,33]. However, Pk is also needed for efficient generation and propagation of polarity, a function hypothesized to occur by negative feedback [19] that appears to occur among clustered complexes. We initially proposed that negative feedback involves the Vang dependent exclusion of [Dsh-Fz-Fmi] complexes [18,19]. However, our current observations suggest that negative feedback might also occur in the opposite direction, in the form of Fz-dependent removal of proximal complexes (Fmi-Vang-Pk) by endocytosis (Figs 6D, 7D, and 9G). We therefore further examined the requirement for Pk in the feedback-dependent amplification/mutual exclusion observed during intercellular PCP communication.

Overexpression of Fz causes hairs in neighboring cells to point away from the clone; this is associated with recruitment of Vang to the boundary of neighboring cells, and at the same time repulsion of Fz from that boundary [30]. Conversely, we observe that overexpressing Vang causes hairs in neighboring cells to point toward the clone, and this is associated with recruitment of Fz and exclusion of Vang from the neighboring cell border (Fig 9I). Because Pk appears to act via Vang, we tested whether Pk might be required for this exclusion of Vang in the wildtype neighbor. We observe that whereas in wildtype cells adjacent to a Vang overexpressing clone, Vang is excluded from the cell boundary, in *pk* mutant cells Vang exclusion was not observed (Fig 9K 9L, and 9M). In contrast, Pk is not required for recruitment of Vang adjacent to cells that express Fz but not Vang (*vang* mutant clone) (S7 Fig). These results support the idea that Pk regulates Vang removal.

## Discussion

### Ubiquitinylation of Pk by the Cul1 E3 ligase complex

In this study, we have shown that Cul1 complex-mediated ubiquitinylation of Pk is required for correct function of the core PCP signaling module, thereby ensuring proper alignment of hairs on the *Drosophila* wing. Ubiquitinylation by the Cul1 complex targets Pk for proteasome-dependent degradation, and in its absence, excess Pk accumulates, resulting in disruption of core PCP function. In several previous reports, ubiquitinylation has been recognized to regulate PCP signaling. In a mouse model, Smurf E3 ligases were shown to regulate PCP signaling by modulating Pk levels [36]. However, mutation of *Drosophila smurf* failed to show PCP defects [37]. In *Drosophila*, Cul3 E3 ligase-BTB protein-mediated regulation of Dsh ubiquitinylation modulates PCP signaling, as does the de-ubiqutinylating enzyme Faf, possibly acting on or upstream of Fmi [37], or more recently proposed to act on Pk [50]. Loss of either activity shows subtle effects on final PCP outcomes in *Drosophila*. In no case is there a demonstrated mechanism for how these events impact the characteristic asymmetric subcellular localization of PCP proteins that underlies cell polarization.

We find that Slimb is the F-box protein that mediates Pk and Cul1 complex association in vivo. It appears likely that the motif that mediates interaction between Pk and Slimb resides in the C-terminal half of the protein, as do the Vang interaction domain and the farnesylation (CaaX) motif [52]. Of note, the amount of Slimb protein in the cell was also dependent on Pk (Fig 4A). In previous cell culture studies, F-box proteins themselves were targeted for ubiquitinylation by their own Cul complexes when not bound by other substrates [48], and this appears to be the case here, as Slimb levels are increased in *cul1* knock-down clones (S3D Fig). Furthermore, this result supports the idea that Pk is the major target of the Cul1 complex during pupal wing development.

If the Cul1-SkpA-Slimb complex targets Pk for degradation, why do Slimb and Pk accumulate together on the proximal side of wildtype cells? Pk is known to bind to Vang, and to localize with it in the proximal complex. Slimb adapts the Cul1 complex to Pk and is seen to colocalize with Vang on the proximal side, as well as with overexpressed Pk (Fig 4). However, this suggests that the Pk in this location is resistant to Cul1 complex-dependent degradation. Pk levels have long been known to be limited by a Vang-dependent activity [33]. Recently, Strutt et al. showed that farnesylation of Pk is required for Pk to interact with Vang and promote its degradation, and that levels of Pk also depend on SkpA, leading to the suggestion that farnesylation-dependent Pk-Vang interaction results in SkpA-dependent Pk degradation [38]. We provide evidence suggesting that the Cul1-SkpA-Slimb E3 complex directly targets Pk for destruction, but in contrast, our finding that Pk with deleted CaaX domain accumulates to elevated levels in *cul1* knock-down cells (S4C and S4D Fig) indicates that Cul1/SkpA/Slimb-dependent degradation is independent of farnesylation. Furthermore, our finding that Pk promotes internalization of Fmi-Vang-Pk during mutual exclusion of oppositely oriented core PCP complexes leads to a model, described below, that is consistent with our shared observation that Pk associated with stable intercellular complexes ([Dsh-Fz-Fmi]-[Fmi-Vang-Pk]) is protected from degradation.

### Generation of cell polarity

**Fig 10:**
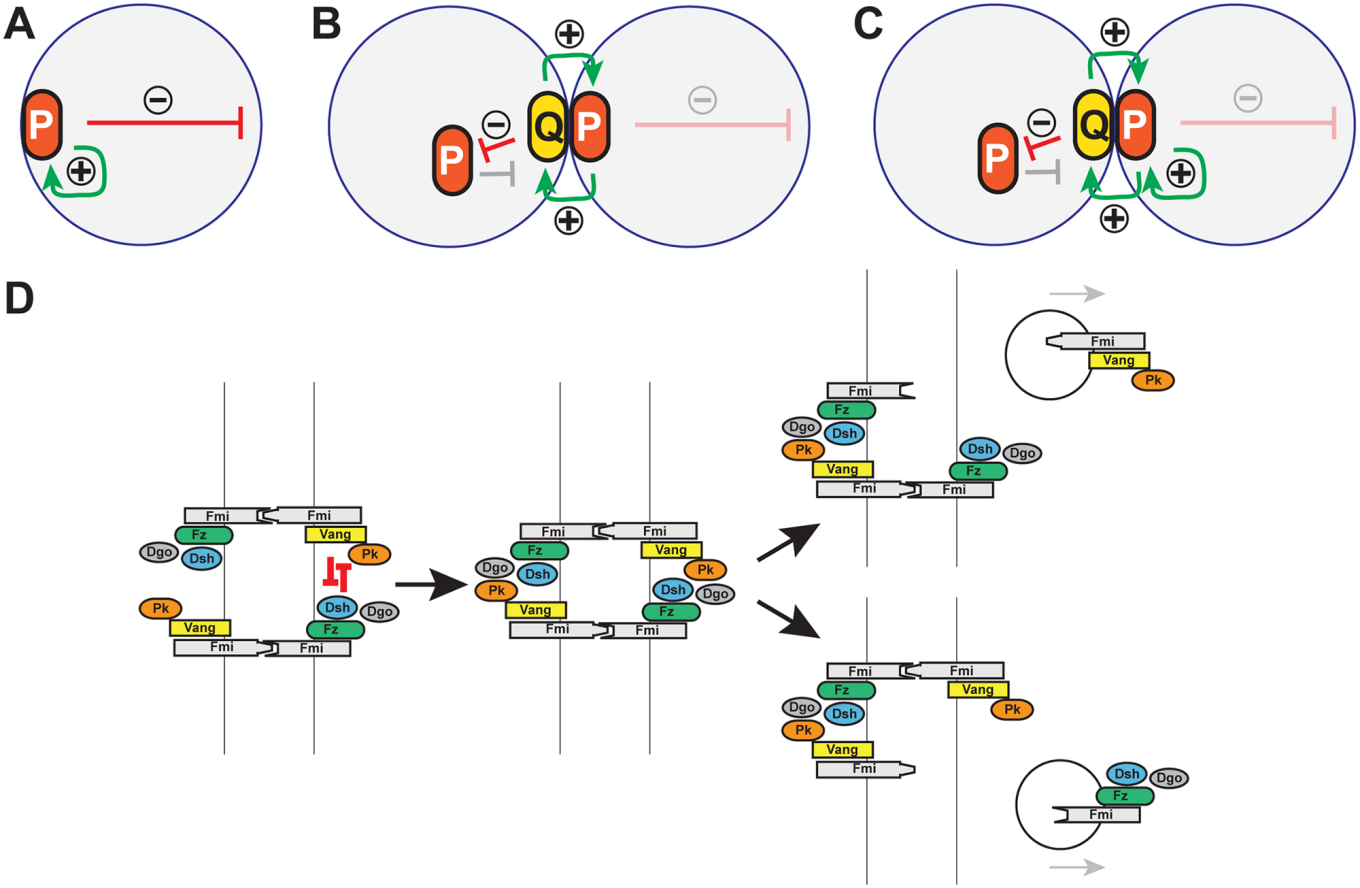
Models: Cell polarity establishment and the involvement of Pk-mediated endocytosis. (A-C) Schematics of positive (green arrows) and negative (red arrows) feedback in polarization of single cells (A), and coupled cells (B, C). Two possible modes of positive feedback are shown in (C). (D) Model of mutual exclusion in which competitive interactions between peripheral membrane associated core factors results in endocytosis of either Vang-Fmi or Fmi-Fz complexes.

In theory, generation of cell polarity requires the combination of a local self-enhancement of a cell polarity factor and a long range inhibition of the same factor [29]. In isolated cells, likely the evolutionarily more ancient mechanism, intracellular local self-enhancement can arise through cooperativity among P proteins (Fig 10A). Intracellular long range inhibition is most easily accomplished by limiting amounts of a component of the P complex, such that aggregation of P complexes in one location decreases the probability of aggregation elsewhere by depletion of that component.

Cell polarization within a multicellular system introduces additional possible intercellular mechanisms for both the local self-enhancement and the long range inhibition (Fig 10B and 10C). If two polarity complexes, P and Q, exist, and can interact at junctions between adjacent cell boundaries, then both the local and long range effects can be mediated through these intercellular interactions. If P complexes recruit Q complexes to opposing sides of junctions, and if mutual antagonism between P and Q occurs, then long range inhibition can occur by P recruiting Q to the neighbor, where P is then excluded (Fig 10B). Similarly, exclusion of P decreases Q in that region of the original cell, enabling the accretion of more P (in effect, cooperativity) (Fig 10C).

The peripheral membrane associated core PCP proteins Pk, Dsh and Dgo appear to mediate these polarization events, but how they do so is not known. They are not required for assembly of asymmetric [Fz-Fmi]-[Fmi-Vang] complexes, but were known to share the ability to induce clustering, and are all required for the feedback amplification that results in the asymmetric subcellular localization of PCP signaling complexes. While their action somehow promotes the assortment of proximal and distal core proteins to opposite sides of the cell, how they carry out this function, and in particular whether this is through intracellular or intercellular mechanisms, is unclear.

### Pk-dependent endocytosis and amplification of PCP

To understand how excess Pk resulting from mutation of the Cul1 E3 complex disrupts PCP, we further studied Pk’s role in establishment of core asymmetry. *pk* mutation causes symmetric distribution of other core proteins without substantially diminishing or enhancing their junctional recruitment. On the other hand, Pk overexpression causes both accumulation of higher levels of all proximal and distal core proteins and induces their clustering at apical membrane domains, generating discrete puncta. A Pk induced clustering of similarly oriented core complexes, as previously proposed [32], could explain both the aggregated punctate appearance and the increased levels of accumulated proteins if one assumes a steady state relationship between free asymmetric complexes and unassembled components as asymmetric complexes are sequestered into puncta.

Our Pk over-expression study shows that Fz is not required for making Fmi clusters, but Vang is (Fig 6). This suggests an intracellular mechanism in which Pk interacts with Vang at the apical membrane to induce clustering. However, since Vang over-expression does not cause accumulation of other core proteins [33], a specific function for Pk beyond stabilization of Vang must be considered to explain the accumulation of other core proteins. Furthermore, the depletion of Fmi from the membrane achieved by the very high levels of Pk upon simultaneous Pk overexpression and Cul1 depletion (S5B, S5C, and S5D Fig) argues for a function for Pk beyond clustering.

### A model for Pk in amplification of core protein asymmetric localization

Pk might stimulate amplification simply by promoting clustering, with long range inhibition mediated by other mechanisms, or perhaps by limiting amounts of Pk. However, our data suggest an alternative interpretation, as we observe Pk-dependent mutual exclusion of oppositely oriented complexes: forcing local accumulation of distal proteins induced the Pk-dependent removal of proximal proteins within the same cell (Fig 9G, 9I, 9K, 9L, and 9M). Exclusion is associated with Pk mediated internalization of Pk-Vang-Fmi complexes, suggesting that this exclusion involves endocytosis (Fig 10D; Pk positive vesicle). The requirement for Vang in this internalization is consistent with a previous study showing that Vang contributes to Fmi internalization [47]. We therefore propose that Pk is involved in an intercellular long range inhibition to promote feedback amplification (Fig 10D).

Like clustering, the Pk-induced routing of Fmi into intracellular vesicles was dependent on Vang, and Pk, Vang and Fmi colocalize in vesicles both apically and more basally (Figs 6 and 7), indicating that Fmi-Vang complex trafficking is regulated by associated Pk. However, unlike clustering, it is also dependent on Fz (Figs 6D and 7D). This suggests a model for feedback inhibition in which oppositely oriented asymmetric complexes interact within clusters, leading to endocytosis and removal of Pk-Vang-Fmi. Competitive interaction between the proximal protein Pk and the distal protein Dgo for Dsh binding is known to occur [19,52–54], suggesting that these interactions might result in either of two alternative outcomes, one of which would be disruption of the proximal complex, and the other disruption of the distal complex (Fig 10D). We propose that if the distal complex ‘wins,’ thus remaining stable, the proximal Pk-Vang-Fmi complex becomes internalized in a Pk-dependent step. Once there is a predominance of complex in a given orientation, Vang will be enriched on one side of the intercellular boundary with relatively little Fz present. Since Pk and Slimb associate with Vang, they too will be enriched, but the absence of competitive interactions from the Fz complex allows them to remain within clusters, accounting for the accumulation of Pk and Slimb on the proximal side of wildtype wing cells. According to this model, Pk and Slimb are observed primarily at sites where they are inactive and therefore not internalized.

Modest levels of Pk overexpression both enhance accumulation of PCP protein complexes at the membrane and disrupt the normal orientation of polarization. This may be explained by enhanced feedback amplification that overwhelms the ability to interpret directional inputs. In contrast, the depletion of Fmi from the membrane observed with the very high levels of Pk induced by simultaneous Pk overexpression and Cul1 depletion (S5B–S5D Fig) suggests that sufficient Pk can induce internalization even without the competitive interactions from the Fz complex that normally stimulate internalization.

The mechanism for Pk-dependent clustering is not known. As previously proposed, clustering may result from a scaffolding effect; the possibility of decreased endocytosis accounting for clustering was previously discounted [32]. Whatever the mechanism, clustering by Pk must occur independent of Fz (Fig 6D). Furthermore, Pk must enable the multimeric aggregation of complexes containing [Vang-Fmi]-[Fmi] or [Vang-Fmi]-[Fmi-Fz]. Induction of multimeric clustering would also provide a context for the dose-dependent competition that determines internalization of either the proximal or distal complex. Additional work will be required to determine how Pk facilitates clustering.

### Function of the Cul1 complex

Since Cul1 depletion increases the amount of Pk, and excess Pk produces clustering and amplification, we now consider how Cul1 might produce the observed phenotype. The simplest possibility is that in the Cul1 mutant, excess Pk produces excess clustering and amplification that overwhelms the directionality in the system. However, because Pk is associated with Slimb and yet stable in the polarized state, and because Pk degradation is dependent on Vang, we also entertain the possibility that Cul1-dependent degradation is somehow functionally coupled to Pk-mediated internalization. Additional studies will be required to distinguish these possibilities.

In summary, we propose a model in which Pk-dependent internalization of proximal complexes provides an intercellular long range inhibition that contributes to amplification of core protein asymmetric localization (Fig 10D). At the same time, Pk provides a local cooperative effect by inducing clustering and accumulation of proximal complexes. We don’t know the mechanism for clustering, but a simple model is that Pk mediates closely related internalization events.

We note that a similar intercellular long range inhibition was initially discussed long ago [18–20], except that [Vang-Pk] was proposed to disrupt [Fz-Dsh]. This interpretation was based largely on inference. Here, we provide evidence that [Fz-Dsh] disrupts [Vang-Pk] (by promoting internalization). On theoretical grounds, either one would be sufficient to cause polarization, but we don’t exclude the possibility that both may occur. Indeed, vesicles containing Fz, Dsh and Fmi have been shown to be transcytosed in a microtubule-dependent fashion with a directional bias [26–28], and these vesicles appear to derive from apical junctions, where they may arise by exclusion.

### Smurf and Slimb

Although knock-down of *smurf* in flies reveals no function in PCP, the mechanism we describe is similar to that inferred for Smurf in mouse PCP [36]. Narimatsu and colleagues found that mice mutant for both Smurf1 and Smurf2 show PCP defects and lose asymmetric localization of core PCP proteins. Furthermore, biochemical evidence was provided that Smurfs, in the presence of the Dsh homolog Dvl2 (and Par6) mediated ubiquitinylation of mouse Pk1. From this, they proposed the model that proximal complexes containing Pk1, and presumably Vang and Celsr (Fmi), are disrupted upon proximity to distal complexes containing Fzd and Dvl2. This model is similar to our model of mutual exclusion, except that the mode of disruption was not directly addressed. While we propose disruption by internalization, perhaps coupled to degradation, they were only able to address degradation. Furthermore, it is not known if, in mouse, Pk1 mediates clustering, perhaps by a related mechanism, as we describe in flies.

The de-ubiquitinase USPX9 was recently identified as a regulator of Pk in the context of Pk’s role in epilepsy in human, mouse, zebrafish and flies [50]. Similarly, the orthologous *Drosophila* de-ubiquitinase Faf modulates the *pk* ^*sple*^ dependent seizure phenotype in flies. These observations suggest that while the ubiquitinylating and de-ubiquitinylating activities of Smurf and USPX9 control the ubiquitinylation state of vertebrate Pk’s, Cul1 and Faf may serve the analogous function to regulate ubiquitinylation of *Drosophila* Pk.

## Materials and Methods

### Fly strains and genetics

All flies were grown at 25°C. The following alleles and stocks were used. FlyBase and VDRC id numbers, when available, are in parentheses. Detailed chromosomes and genotypes are provided in the figure legends. *UAS-cul1* ^*RNAi*^ (VDRC# 108558, 42445); *UAS-skpA* ^*RNAi*^ (VDRC# 32789, 107815); *UAS-slimb* ^*RNAi*^ (FBst0033898, FBst0033986); *UAS-fmi* ^*RNAi*^ (FBst0026022); *FRT42D cul1* ^*EX*^ (from R. Duronio, UNC, Chapel Hill, USA), *pk* ^*pk-sple13*^ [12]; *FRT42D pk* ^*pk-sple13*^ (FBst0044230); *pk* ^*pk-sple14*^ [12]; *pk* ^*pk30*^ (FBst0044229); *pk* ^*sple1*^ (FBst0000422); *vang* ^*A3*^ [10]; *FRT42D vang* ^*A3*^ [10]; v*ang* ^*stbm6*^ (FBst0006918); *fz* ^*R52*^; *actP>CD2>GAL4 UAS-GFP* (from B. Lu, Stanford, Stanford, USA); *actP>CD2>GAL4 UAS-RFP* (FBst0030558); *D174GAL4* [27]; *actP>CD2>GAL4* (FBst0004779); *ptc-GAL4* (FBst0002017); *armP-fz*::*GFP* [30]; *Cas-dsh*::*GFP* [27]; *actP-vang*::*YFP*; *UAS-GFP*::*pk* [12]; *UAS-GFP*::*sple* [12]; *UAS-pk* ^*pk*^ [12]; *tubP-6XMyc*::*slimb* (from E. Verheyen, Simon Fraser University, Burnaby, Canada); *FRT42D ubiP-NLS*::*mRFP* (FBst0035496); *UAS-fmi::YFP [30]*; *UAS-vang* [30]; *actP-GFP*::*pk* ^*dCaaX*^ and *actP-6XMyc*::*pk* (from D. Strutt, University of Sheffield, Sheffield, UK); *UAS-Dcr2* (FBst0024646); *FRT80B* (FBst0001988); *UAS-lacZ* ^*RNAi*^ (R. Carthew, Northwestern University, Evanston, USA); *UAS-prosbeta2’* (FBst0006785).

C-terminal deleted *pk* (aa1-472) was tagged at the N-terminus with *HA* (YPYDVPDYA) and cloned into pUASt vector (S1 Text).

*vang*::*EYFP*, *vang*::*ECFP*, *fz*::*ECFP* and *ECFP*::*dgo* were generated by fusing the respective fluorophore at the 5’ or 3’ end of the corresponding cDNA. *vang*::*ECFP* and *fz*::*ECFP* carry an additional *HA*-tag sequence between the respective coding sequences. Sequences were cloned into pCM43-ubiP-SV40 [55] and integrated into the VK0033 landing site by target-site specific transgenesis [56,57].

Transgenic flies were generated by BestGene Inc. (Chino Hills, CA, USA) for the C-terminal deleted *pk* construct and direct injection for other constructs.

FLP-on (using the *actP>CD2>GAL4* construct for trans-gene expression) and FLP/FRT mitotic clones were generated by incubating third-instar (for analyzing pupal wings) or second-instar (for analyzing wing discs) larvae at 37°C for 1 hr and pupal wings (60 to 72 hr after heat shock) and third-instar larval wing discs (48 hr after heat shock) with appropriate clones were selected for analysis at indicated developmental time points.

### Immunohistochemistry

Primary antibodies were as follows: mouse monoclonal anti-Fmi (1:200 dilution, DSHB), guinea pig polyclonal anti-Pk (1:800, [27]), rat monoclonal anti-HA (clone 3F10, 1:200 dilution, Roche), rabbit polyclonal anti-c-Myc (1:200 dilution, Santa Cruz), mouse anti-LacZ (1:500 dilution, Promega). Secondary antibodies from Life Technologies were as follows: 488-donkey anti-mouse, 488-donkey anti-rabbit, 594-donkey anti-mouse, 633-goat anti-guinea pig, 633-goat anti-mouse, 633-goat anti-rat, 647-goat anti-rabbit. Alexa 635 and Alexa 488 conjugated phalloidin were from Life Technologies.

### Imaging and quantification

As mutation or knock-down of components in the Cul1 complex induces size and shape defects of cells, clonal wings with mild defects where cell shape and size are comparable with wildtype cells were selected for quantification. To measure the intensity of junctional core proteins in Fig 2 and S1 Fig, 50 P/D and 20 A/P junctions were randomly selected from *cul1i* clones and wildtype areas from each of five wings. Using Adobe Photoshop, mean intensity was obtained for P/D and A/P junctions and a background intensity from the non-junctional region was subtracted. Fold differences were calculated for each wing. Average fold differences were calculated and p-values were obtained from a t-test with values obtained from five wings for each genotype.

Vesicle numbers were quantified by imaging through several planes and counting vesicles/cell in the relevant wildtype and mutant cells. Since this does not capture a complete apical-basal scan, numbers were normalized against the wildtype value to allow comparisons between mutant and wildtype, and expressed as a percentage for each image stack.

To analyze adjacent clones expressing ECFP and EYFP-tagged PCP proteins in Fig 9, pupae were dissected at 16 hAPF as previously described [58]. Pupal wings were imaged using an Olympus Fluoview FV1000 confocal microscope equipped with a 63x oil immersion lens. ECFP and EYFP were excited using 458 and 514 nm laser lines, respectively. Boundary profiles were measured in Fiji using a 3px wide line and the Plot Profile function. To plot normalized boundary profiles for the adjacent Vang::ECFP and Vang::EYFP twinspots, measured boundary intensities were normalized to the average pixel intensity along cell boundaries that only touch cells belonging to the respective twinspot. All other immunofluorescence images were taken with a Leica TCS SP5 AOBS confocal microscope and processed with LAS AF (Leica) and Adobe Photoshop. Adult wings were imaged with Spot Flex camera (Model 15.2 64 MP) equipped with Nikon Eclipse E1000M.

### FM4-64 uptake assay

Live pupae with partially ripped cuticle were incubated in 10μg/ml FM4-64 (Life Technologies) in M3 media (Sigma-Aldrich) for 15-30m at 25°C. After the incubation, wings were directly subjected to confocal microscopy.

### Western blot and immunoprecipitation

Third-instar larval wing discs were dissected and lysed in protein loading buffer. Lysates from eight discs were loaded per lane for SDS-PAGE analysis. Antibodies: Guinea pig polyclonal anti-Pk (1:1000), mouse monoclonal anti-Myc (1:1000, Sigma-Aldrich), mouse monoclonal anti-γ-Tubulin (1:1000, Sigma-Aldrich). For the experiments using the temperature sensitive dominant negative form of proteasomal subunit, Prosbeta2’, larvae with appropriate genotypes were incubated for 2hr 30 min at 30°C before isolating wing discs. Lysates from 15 wing discs were loaded in each lane.

To immunoprecipitate Myc-tagged Pk, 80 wing discs from each genotype, grown under the same condition as for the Western blot assay, were lysed and ground in 50μl of lysis buffer (2% SDS, 150mM NaCl, 10mM Tris-HCl, pH 7.5) with 2mM sodium orthovanadate, 50 mM sodium fluoride, and protease inhibitors. After boiling samples for 5 min, 350μl of dilution buffer (150mM NaCl, 10mM Tris-HCl, pH 7.5, 2mM EDTA, 1% Triton) was added and samples were incubated at 4°C for 30min (ref. modified from [59]). Samples were spun at 15,000 x g for 5 min, and anti-Myc affinity gel (Biotool) was added to the supernatants and the mixtures were incubated at 4°C for 3 hr. The remaining immunoprecipitation procedures were as described by the Biotool manual. Rabbit polyclonal anti-Ubiquitin antibody (1:2000, Thermo Scientific) was used to detect ubiquitinylated Myc::Pk.

## Supporting Information

S1 Fig Requirement of the Cul1 complex for core PCP control. Enrichment of Dsh::GFP (A; green in A’) and Vang::YFP (B; green in B’) at the apical junction in *cul1* knock-down clones (RFP positive in A’, B’) at 28hr APF. (A”, B”) fold differences of signal intensity for each core protein at junctions between *cul1* RNAi or wildtype cells (***P<0.0001; t-test). Apical Fmi (C and E; red in C’ and E’) and Pk (D and F; blue in D’ and F’) were labelled in wing tissues harboring *skpA* (C, D) or *slimb* (E, F) knock-down clones (GFP) at 28hr APF. Apical staining of Fmi or Pk is enriched in *skpA* and *slimb* knock-down cells, similar to that seen in *cul1* knock-down cells (Fig 2). Scale bars: 10μm. Genotypes are (A) *y*, *w*, *hsflp/+; UAS-cul1* ^*IR108558*^ */Cas-dsh*::*GFP; actP>CD2>GAL4*, *UAS-RFP/+*, (B) *y*, *w*, *hsflp/+; UAS-cul1* ^*IR108558*^ */actP-vang*::*YFP; actP>CD2>GAL4*, *UAS-RFP/+*, (C, D) *y*, *w*, *hsflp/+(Y); UAS-skpA* ^*IR32789*^ */+; actP>CD2>GAL4*, *UAS-GFP/+*, (E, F) *y*, *w*, *hsflp/+(Y); +/+; actP>CD2>GAL4*, *UAS-GFP/UAS-slimb* ^*IRFBst0033898*^.
(TIF)

S2 Fig Cul1 complex-mediated control of Pk protein is Fz-independent and post-transcriptional. *cul1* knock-down clones (marked with GFP; green in A”) were introduced in *fz* mutant (*fz* ^*R52*^ */fz* ^*R52*^) wings. Within the clones, clustering of apical Pk (A; blue in A”) and Fmi (A’; red in A”) is seen (28hr APF). *cul1* mutant (*cul1* ^*EX*^) clones (no RFP; B’, C’, D’ and E’) were generated in *D174GAL4*; *UAS-GFP*::*pk* third instar wing discs (B, C) and wings at 24hr APF (D, E). *cul1* clones accumulate exogenously driven GFP::Pk in third instar wing discs (B, C) as well as pupal wings at 24hr APF (D, E). Scale bars: 75μm (B, C), 10μm (D, E). Genotypes are (A) *y*, *w*, *hsflp/+; UAS-cul1* ^*IR108558*^ */ actP>CD2>GAL4*, *UAS-GFP; fz* ^*R52*^ */*fz^R52^, (B-E) *y*, *w*, *hsflp/D174GAL4; FRT42D*, *cul1* ^*EX*^ */FRT42D*, *ubiP-NLS*::*mRFP; UAS-GFP*::*pk/+*.
(TIF)

S3 Fig Accumulation and localization of apical Slimb depends on core proteins and on Cul1. Ability of Fmi or Vang to modify Slimb localization was tested in *tubP-6XMyc*::*slimb* fly wings (A and B, 28hr APF). Myc::Slimb (A’, B’) patterns visualized with anti-c-Myc antibodies (blue in A” and B”) in- and outside clones overexpressing *fmi*::*YFP* (green, A”) and *vang* (RFP, B”) (outlined in A’ and B’). Overexpression of *vang*, but not *fmi*::*YFP*, induces modestly enhanced apical Slimb localization within overexpression domains. (C) Myc::slimb and Pk expression (C’) were monitored in 28hr APF wings bearing *slimb* knock-down clones. *slimb* knock-down clones abolished Myc::Slimb labeling where Pk accumulates, showing antibody specificity. Regions surrounding *slimb* knock-down clones show domineering non-autonomy (C, or C”c for magnified images), where apical Myc::Slimb (green) is coordinately re-localized with Pk (red), although Myc::slimb localization is considerably less asymmetric and membrane associated (see also Fig 4). (D) *cul1* knock-down clones (RFP in D’) accumulate Myc::Slimb (blue, D’) in apical (D) and basal planes (D”) at 28hr APF, suggesting that the retention of Slimb is also dependent on the Cul1 complex. Scale bars: 10μm. Genotypes are (A) *y*, *w*, *hsflp/+; tubP-6XMyc*::*slimb/+; actP>CD2>GAL4*, *UAS-RFP/UAS-fmi*::*YFP*, (B) *y*, *w*, *hsflp/+; tubP-6XMyc*::*slimb/+; actP>CD2>GAL4*, *UAS-RFP/UAS-vang*, (C) *y*, *w*, *hsflp/+(Y); tubP-6XMyc*::*slimb/tubP-6XMyc*::*slimb; actP>CD2>GAL4*, *UAS-RFP/UAS-slimb* ^*IRFBst0033898*^, (D) *y*, *w*, *hsflp/+; tubP-6XMyc*::*slimb/ UAS-cul1* ^*IR108558*^ *; actP>CD2>GAL4*, *UAS-RFP/+*.
(TIF)

S4 Fig Requirement of CaaX motif and C-terminus of Pk for Cul1-mediated control and Vang localization. *ptc-GAL4* driven *pk* induces apical accumulation and clustering of Vang::YFP (A’; green in A”‘) and Fmi (A”; blue in A”‘) (A). However, when Fmi is simultaneously knocked down (using *UAS-fmi* ^*IRFBst0026022*^ and *UAS-Dcr2* in the same genetic background, B”), Vang::YFP accumulates apically (B’) but does not show the same clustering pattern. Pk (red) in A”‘ and B”‘. A, B; 28hr APF. GFP::Pk^dCaaX^ accumulates in *cul1* knock-down clones (C). *cul1* knock-down clones (RFP; C” and D’) were generated in *actP-GFP*::*pk* ^*dCaaX*^ wings and GFP::Pk^dCaaX^ (C and D; green in C”, D’) and Fmi (C’; blue in C”) were monitored (C and D; 28 hr APF). GFP::Pk^dCaaX^ localization is enriched at cell junctions in *cul1* knock-down clones (C, compare with Fmi patterns in C’) (C, apical; D, sub-apical). The effect of overexpressing Pk lacking its C-terminus on Vang::YFP patterns was analyzed in- and outside *HA*::*pk* ^*dC*^ overexpressing clones in *actP-vang*::*YFP* wing tissues (E and F; 28hr APF). HA::Pk^dC^ was labelled with anti-HA antibodies. Note that apical HA::Pk^dC^ does not localize asymmetrically and is present in apical (E) and basal (F) cytosol. Vang::YFP localization was not affected by *HA*::*pk* ^*dC*^ overexpression (E’ and F’; compare with A’, Figs 6B and 7B). Scale bars: 10μm. Genotypes are (A) *ptc-GAL4/UAS-pk; actP-vang*::*YFP/+*, (B) *UAS-Dcr2/+; ptc-GAL4/UAS-pk; actP-vang*::*YFP/UAS-*fmi^IRFBst0026022^, (C, D) *y*, *w*, *hsflp/actP-GFP*::*pk* ^*dCaaX*^ *; UAS-cul1* ^*IR108558*^ */+; actP>CD2>GAL4*, *UAS-RFP/+*, (E, F) *y*, *w*, *hsflp/+; UAS-HA*::*pk* ^*dC*^ */ actP-vang*::*YFP; actP>CD2>GAL4*, *UAS-RFP/+*.
(TIF)

S5 Fig Very high Pk expression reduces the amount of cellular Fmi. (A) *cul1* ^*EX*^ mutant clones (outlined in A and A’) induce an excess of Pk (A; red in A”) and Fmi (A’; green in A”) double positive vesicles compared to neighboring wildtype tissue. A sub-apical section is shown. (B-D) In wing tissue overexpressing *GFP*::*pk* with *D174GAL4* (as in Fig 3E), *cul1* homozygous mutant (*cul1* ^*EX*^) clones (no RFP in B”, C” and D”) were examined at 26hr APF. Fmi (B’, C’ and D’; blue in B”, C” and D”) expression was monitored by antibody staining. GFP::Pk (green in B”, C” and D”) accumulation in *cul1* mutant clones is robust in apical (B), sub-apical (C), and basal (D) planes. Notably, overall Fmi staining is reduced inside the clones (B’, C’, D’), as compared to cells outside the clones, where *GFP*::*pk* overexpression induces formation of Fmi-positive vesicles and high levels of clustered apical Fmi, as in Figs 6 and 7. Scale bars: 10μm. Genotypes are (A) *y*, *w*, *hsflp/+(Y); FRT42D*, *cul1* ^*EX*^ */FRT42D*, *ubiP-NLS*::*mRFP*, (B-D) *y*, *w*, *hsflp/D174GAL4; FRT42D*, *cul1* ^*EX*^ */FRT42D*, *ubiP-NLS*::*mRFP; UAS-GFP*::*pk/+*.
(TIF)

S6 Fig Vang vesicle induction in cells inside and outside of Pk overexpressing cells. *pk* overexpression (RFP in A’) clusters Vang::YFP at the apical membrane (A). In sub-apical planes (B), Vang::YFP positive vesicles are seen inside the *pk* overexpressing cells (B, RFP for *pk* overexpressing clones in B’) and also in neighboring wildtype cells (arrowheads in the magnified image, Ba). (Bb) A magnified image of the square region in B’. 26hr APF. Scale bars: 10μm. Genotype: *y*, *w*, *hsflp/+; UAS-pk/ actP-vang*::*YFP; actP>CD2>GAL4*, *UAS-RFP/+*.
(TIF)

S7 Fig Pk is not required for sequestering Vang. *vang* mutant clones in the presence of Fz recruit Vang from neighboring cells to the adjacent cell boundary, causing domineering non-autonomy. To assess whether Pk is required in the responding cell for Vang recruitment, we carried out a twinspot assay. *FRT42D*, *vang* ^*A3*^ */FRT42D*, *pk* ^*pk-sple13*^, *actP-vang*::*YFP* (A) flies were used (*vang* mutant clones with Vang::YFP only in surrounding cells); some surrounding cells are wild-type, and others are *pk* mutant twin clones. Pk visualized by Pk staining (A’, red in A”). Yellow dots indicate *pk* mutant twin cells facing *vang* mutant clones (Aa and Ab: magnified images for squares in A). Vang::YFP is recruited to the adjacent membrane of cells abutting *vang* mutant cells regardless of whether they express *pk* (Aa and Ab; magnification of boxed regions in A; compare membranous Vang::YFP facing *vang* mutant cells in cells with and without yellow dots; yellow arrows indicate membranous Vang::YFP domains formed in *pk* mutant cells). 28hr APF. Scale bars: 10μm. Genotype: *y*, *w*, *hsflp/+; FRT42D*, *vang* ^*A3*^ */FRT42D*, *pk* ^*pk-sple13*^, *actP-vang*::*YFP*.
(TIF)

S1 Table Apical patterns of core PCP factors and hair polarity in clonal wing tissues. Clonal mutation or overexpression effects of core PCP components on other core PCP factors at apical junctions, and hair polarities are compared to those resulting from clonal Cul1 complex mutation.
(DOCX)

S1 Text HA-tagged C-terminal deletion construct of pk. Cloning procedures to generate C-terminal deleted *pk* (aa1-472) with N-terminal *HA* (YPYDVPDYA) tag is described.
(DOCX)
