# Supplementary figures and images for: Clustering and Negative Feedback by Endocytosis in Planar Cell Polarity Signaling Is Modulated by Ubiquitinylation of Prickle
Source: PLoS Genet. 2015 May 21;11(5):e1005259. doi: 10.1371/journal.pgen.1005259 (PMC4440771; doi:10.1371/journal.pgen.1005259)

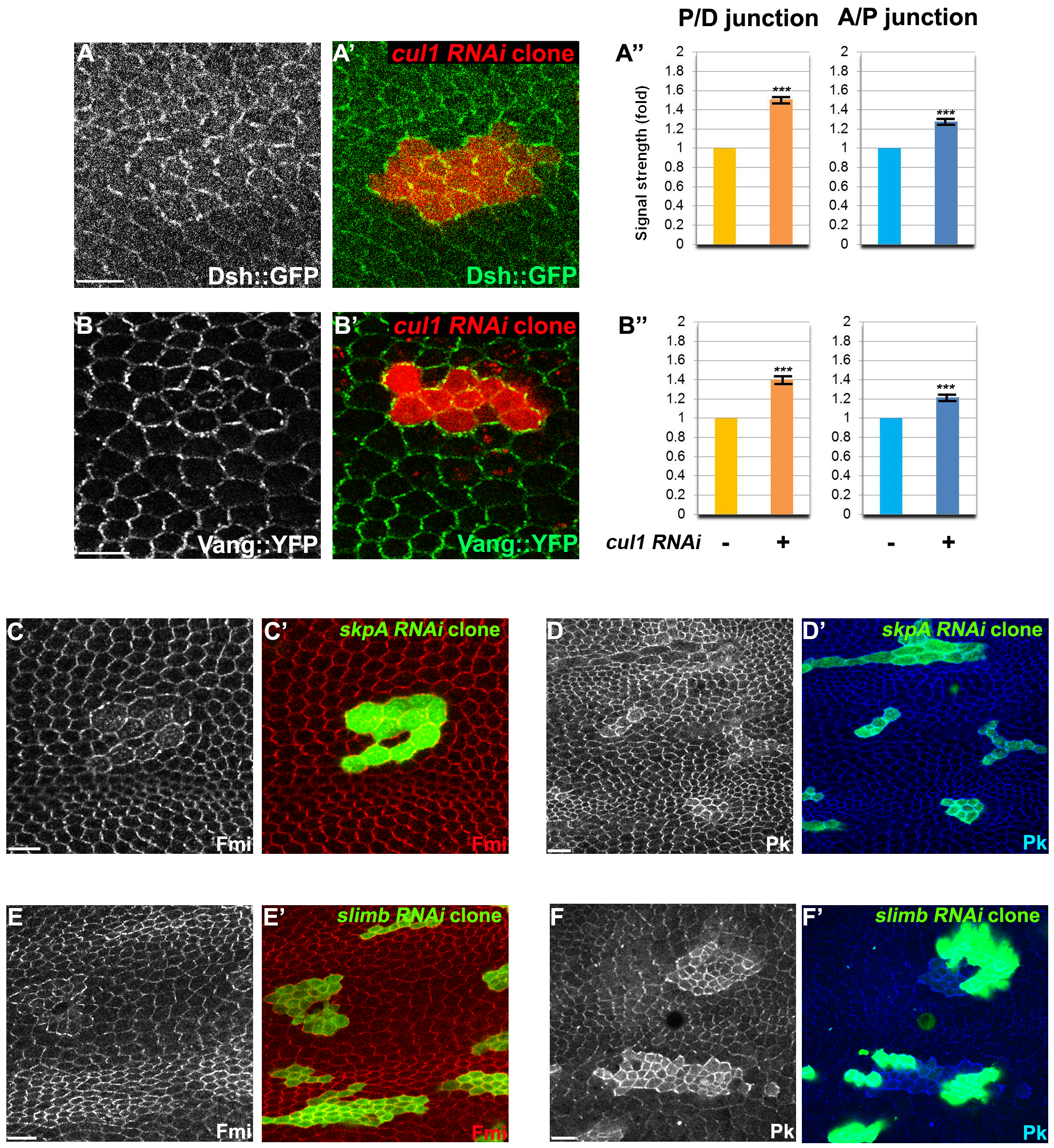

Supplement: S1 Fig — Enrichment of Dsh::GFP (A; green in A’) and Vang::YFP (B; green in B’) at the apical junction in cul1 knock-down clones (RFP positive in A’, B’) at 28hr APF. (A”, B”) fold differences of signal intensity for each core protein at junctions between cul1 RNAi or wildtype cells (***P<0.0001; t-test). Apical Fmi (C and E; red in C’ and E’) and Pk (D and F; blue in D’ and F’) were labelled in wing tissues harboring skpA (C, D) or slimb (E, F) knock-down clones (GFP) at 28hr APF. Apical staining of Fmi or Pk is enriched in skpA and slimb knock-down cells, similar to that seen in cul1 knock-down cells (Fig 2). Scale bars: 10μm. Genotypes are (A) y, w, hsflp/+; UAS-cul1 IR108558 /Cas-dsh::GFP; actP>CD2>GAL4, UAS-RFP/+, (B) y, w, hsflp/+; UAS-cul1 IR108558 /actP-vang::YFP; actP>CD2>GAL4, UAS-RFP/+, (C, D) y, w, hsflp/+(Y); UAS-skpA IR32789 /+; actP>CD2>GAL4, UAS-GFP/+, (E, F) y, w, hsflp/+(Y); +/+; actP>CD2>GAL4, UAS-GFP/UAS-slimb IRFBst0033898. (TIF) [file pgen.1005259.s001.tif]

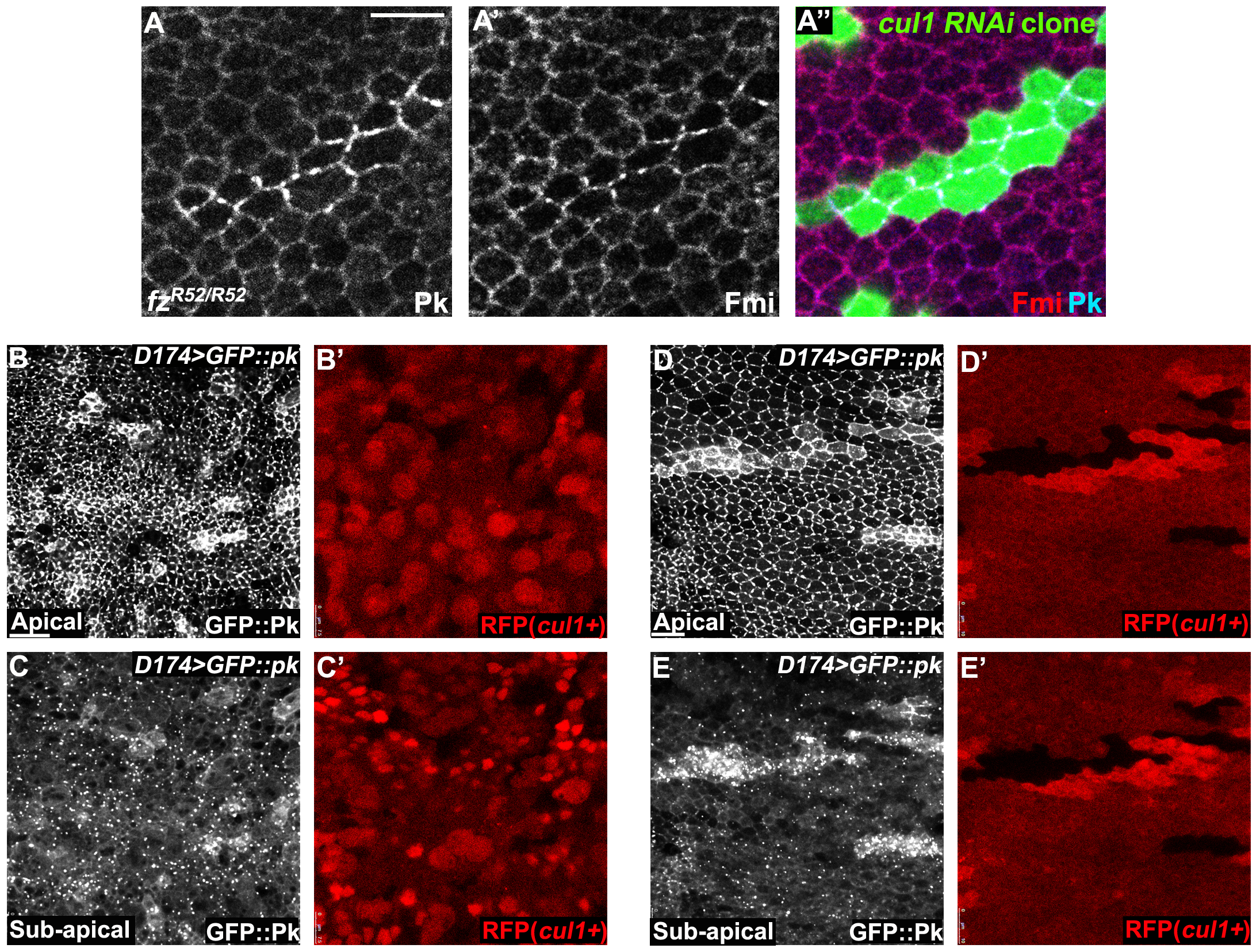

Supplement: S2 Fig — cul1 knock-down clones (marked with GFP; green in A”) were introduced in fz mutant (fz R52 /fz R52) wings. Within the clones, clustering of apical Pk (A; blue in A”) and Fmi (A’; red in A”) is seen (28hr APF). cul1 mutant (cul1 EX) clones (no RFP; B’, C’, D’ and E’) were generated in D174GAL4; UAS-GFP::pk third instar wing discs (B, C) and wings at 24hr APF (D, E). cul1 clones accumulate exogenously driven GFP::Pk in third instar wing discs (B, C) as well as pupal wings at 24hr APF (D, E). Scale bars: 75μm (B, C), 10μm (D, E). Genotypes are (A) y, w, hsflp/+; UAS-cul1 IR108558 / actP>CD2>GAL4, UAS-GFP; fz R52 /fzR52, (B-E) y, w, hsflp/D174GAL4; FRT42D, cul1 EX /FRT42D, ubiP-NLS::mRFP; UAS-GFP::pk/+. (TIF) [file pgen.1005259.s002.tif]

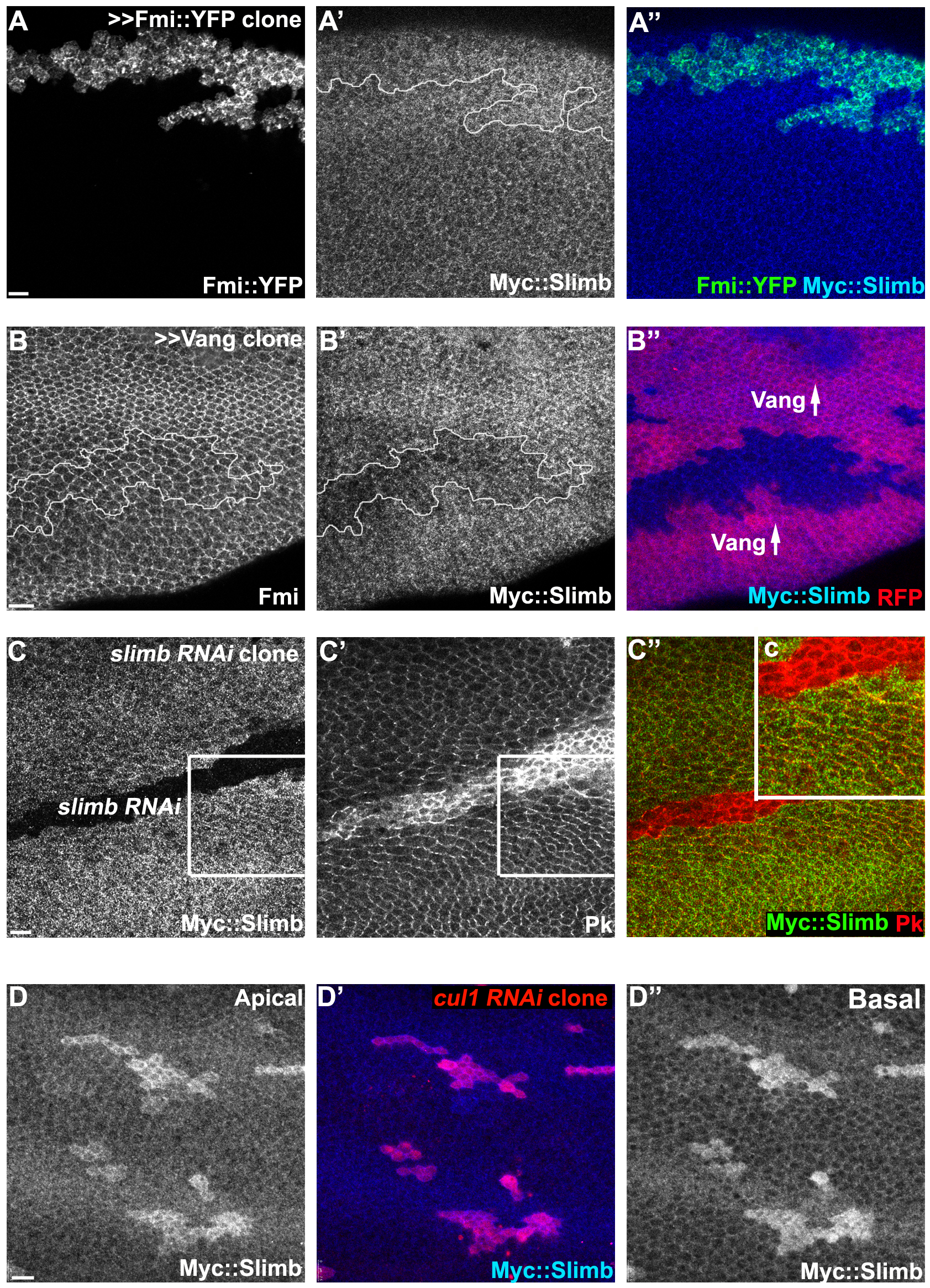

Supplement: S3 Fig — Ability of Fmi or Vang to modify Slimb localization was tested in tubP-6XMyc::slimb fly wings (A and B, 28hr APF). Myc::Slimb (A’, B’) patterns visualized with anti-c-Myc antibodies (blue in A” and B”) in- and outside clones overexpressing fmi::YFP (green, A”) and vang (RFP, B”) (outlined in A’ and B’). Overexpression of vang, but not fmi::YFP, induces modestly enhanced apical Slimb localization within overexpression domains. (C) Myc::slimb and Pk expression (C’) were monitored in 28hr APF wings bearing slimb knock-down clones. slimb knock-down clones abolished Myc::Slimb labeling where Pk accumulates, showing antibody specificity. Regions surrounding slimb knock-down clones show domineering non-autonomy (C, or C”c for magnified images), where apical Myc::Slimb (green) is coordinately re-localized with Pk (red), although Myc::slimb localization is considerably less asymmetric and membrane associated (see also Fig 4). (D) cul1 knock-down clones (RFP in D’) accumulate Myc::Slimb (blue, D’) in apical (D) and basal planes (D”) at 28hr APF, suggesting that the retention of Slimb is also dependent on the Cul1 complex. Scale bars: 10μm. Genotypes are (A) y, w, hsflp/+; tubP-6XMyc::slimb/+; actP>CD2>GAL4, UAS-RFP/UAS-fmi::YFP, (B) y, w, hsflp/+; tubP-6XMyc::slimb/+; actP>CD2>GAL4, UAS-RFP/UAS-vang, (C) y, w, hsflp/+(Y); tubP-6XMyc::slimb/tubP-6XMyc::slimb; actP>CD2>GAL4, UAS-RFP/UAS-slimb IRFBst0033898, (D) y, w, hsflp/+; tubP-6XMyc::slimb/ UAS-cul1 IR108558 ; actP>CD2>GAL4, UAS-RFP/+. (TIF) [file pgen.1005259.s003.tif]

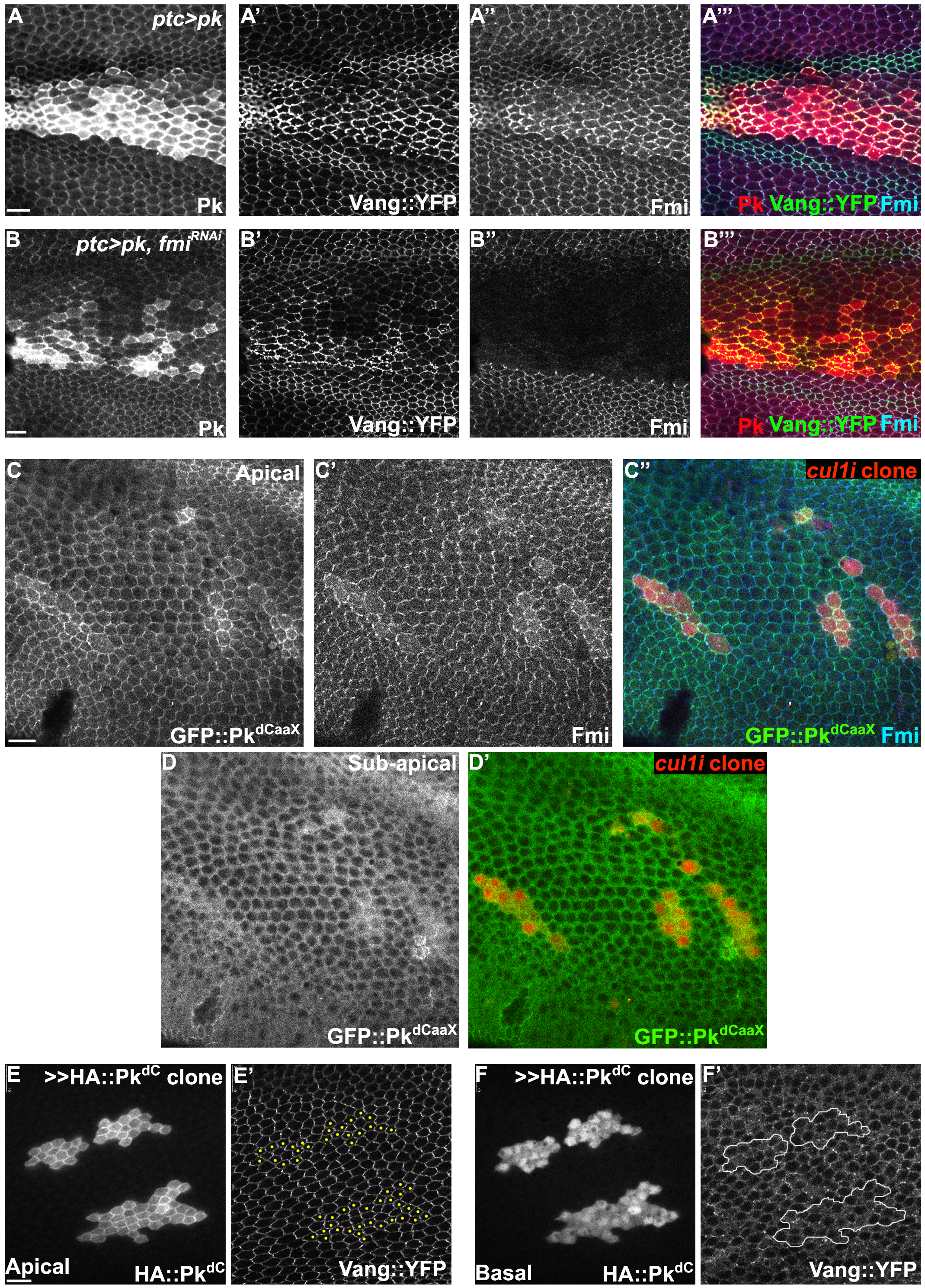

Supplement: S4 Fig — ptc-GAL4 driven pk induces apical accumulation and clustering of Vang::YFP (A’; green in A”‘) and Fmi (A”; blue in A”‘) (A). However, when Fmi is simultaneously knocked down (using UAS-fmi IRFBst0026022 and UAS-Dcr2 in the same genetic background, B”), Vang::YFP accumulates apically (B’) but does not show the same clustering pattern. Pk (red) in A”‘ and B”‘. A, B; 28hr APF. GFP::PkdCaaX accumulates in cul1 knock-down clones (C). cul1 knock-down clones (RFP; C” and D’) were generated in actP-GFP::pk dCaaX wings and GFP::PkdCaaX (C and D; green in C”, D’) and Fmi (C’; blue in C”) were monitored (C and D; 28 hr APF). GFP::PkdCaaX localization is enriched at cell junctions in cul1 knock-down clones (C, compare with Fmi patterns in C’) (C, apical; D, sub-apical). The effect of overexpressing Pk lacking its C-terminus on Vang::YFP patterns was analyzed in- and outside HA::pk dC overexpressing clones in actP-vang::YFP wing tissues (E and F; 28hr APF). HA::PkdC was labelled with anti-HA antibodies. Note that apical HA::PkdC does not localize asymmetrically and is present in apical (E) and basal (F) cytosol. Vang::YFP localization was not affected by HA::pk dC overexpression (E’ and F’; compare with A’, Figs 6B and 7B). Scale bars: 10μm. Genotypes are (A) ptc-GAL4/UAS-pk; actP-vang::YFP/+, (B) UAS-Dcr2/+; ptc-GAL4/UAS-pk; actP-vang::YFP/UAS-fmiIRFBst0026022, (C, D) y, w, hsflp/actP-GFP::pk dCaaX ; UAS-cul1 IR108558 /+; actP>CD2>GAL4, UAS-RFP/+, (E, F) y, w, hsflp/+; UAS-HA::pk dC / actP-vang::YFP; actP>CD2>GAL4, UAS-RFP/+. (TIF) [file pgen.1005259.s004.tif]

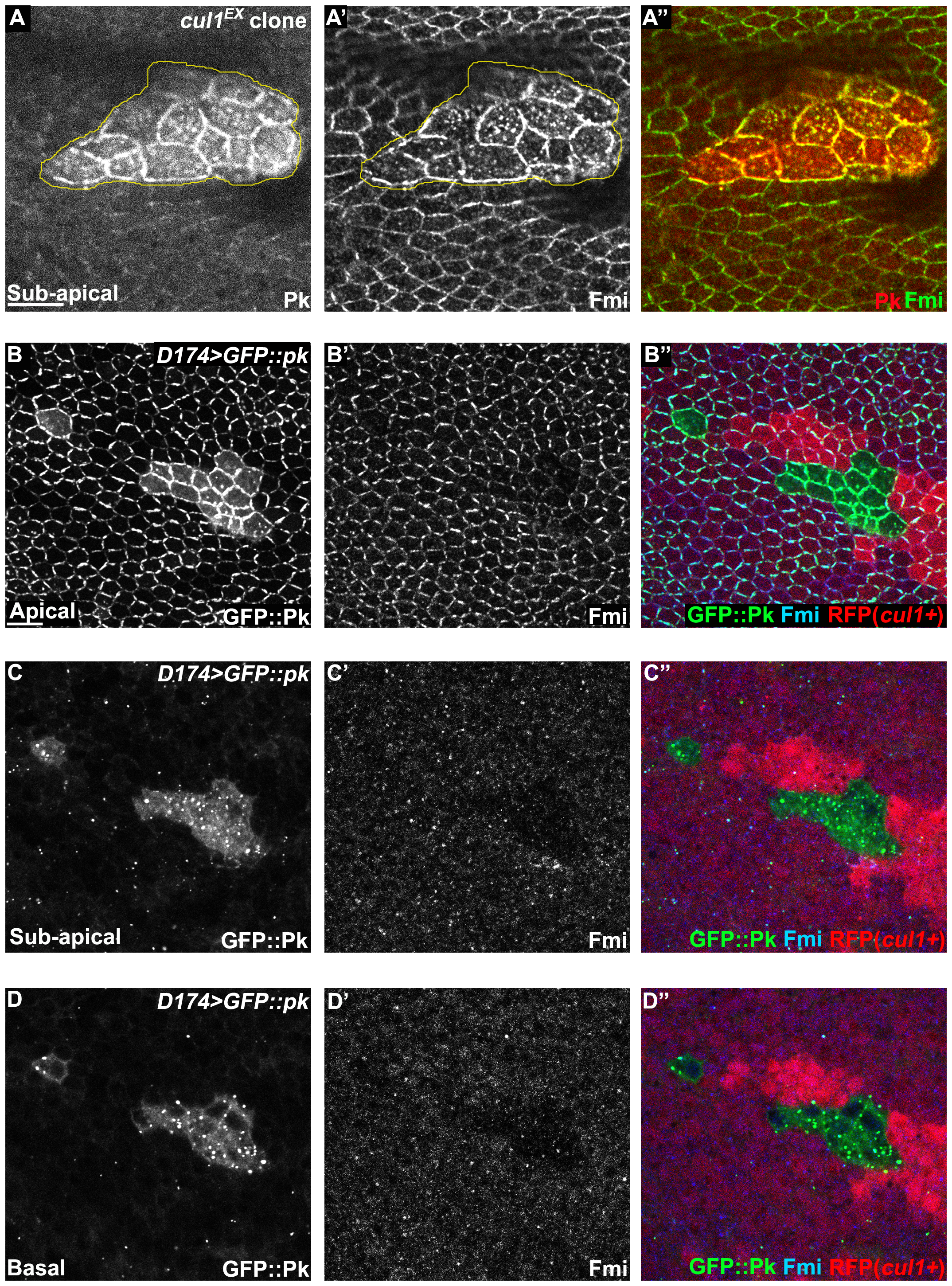

Supplement: S5 Fig — (A) cul1 EX mutant clones (outlined in A and A’) induce an excess of Pk (A; red in A”) and Fmi (A’; green in A”) double positive vesicles compared to neighboring wildtype tissue. A sub-apical section is shown. (B-D) In wing tissue overexpressing GFP::pk with D174GAL4 (as in Fig 3E), cul1 homozygous mutant (cul1 EX) clones (no RFP in B”, C” and D”) were examined at 26hr APF. Fmi (B’, C’ and D’; blue in B”, C” and D”) expression was monitored by antibody staining. GFP::Pk (green in B”, C” and D”) accumulation in cul1 mutant clones is robust in apical (B), sub-apical (C), and basal (D) planes. Notably, overall Fmi staining is reduced inside the clones (B’, C’, D’), as compared to cells outside the clones, where GFP::pk overexpression induces formation of Fmi-positive vesicles and high levels of clustered apical Fmi, as in Figs 6 and 7. Scale bars: 10μm. Genotypes are (A) y, w, hsflp/+(Y); FRT42D, cul1 EX /FRT42D, ubiP-NLS::mRFP, (B-D) y, w, hsflp/D174GAL4; FRT42D, cul1 EX /FRT42D, ubiP-NLS::mRFP; UAS-GFP::pk/+. (TIF) [file pgen.1005259.s005.tif]

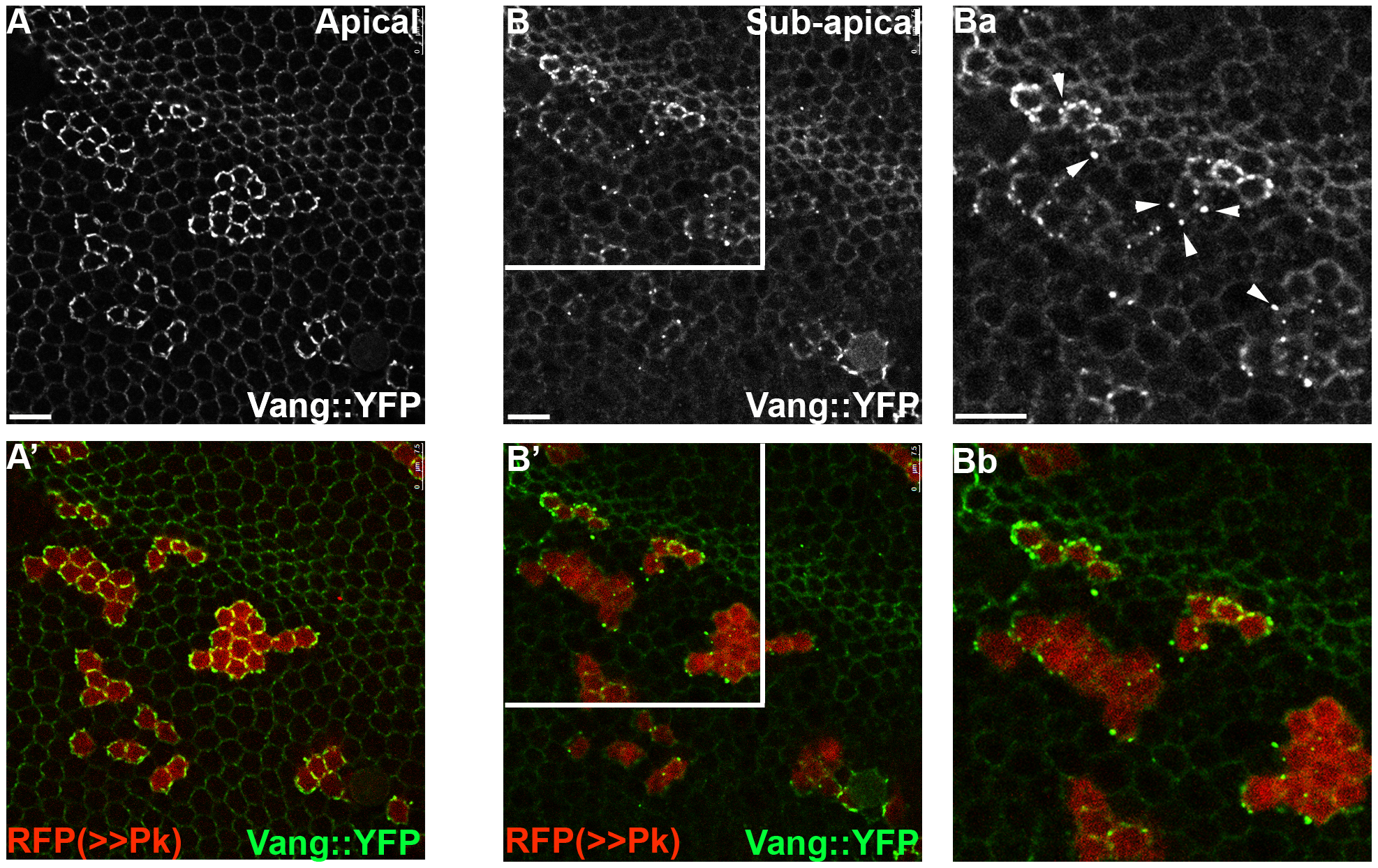

Supplement: S6 Fig — pk overexpression (RFP in A’) clusters Vang::YFP at the apical membrane (A). In sub-apical planes (B), Vang::YFP positive vesicles are seen inside the pk overexpressing cells (B, RFP for pk overexpressing clones in B’) and also in neighboring wildtype cells (arrowheads in the magnified image, Ba). (Bb) A magnified image of the square region in B’. 26hr APF. Scale bars: 10μm. Genotype: y, w, hsflp/+; UAS-pk/ actP-vang::YFP; actP>CD2>GAL4, UAS-RFP/+. (TIF) [file pgen.1005259.s006.tif]

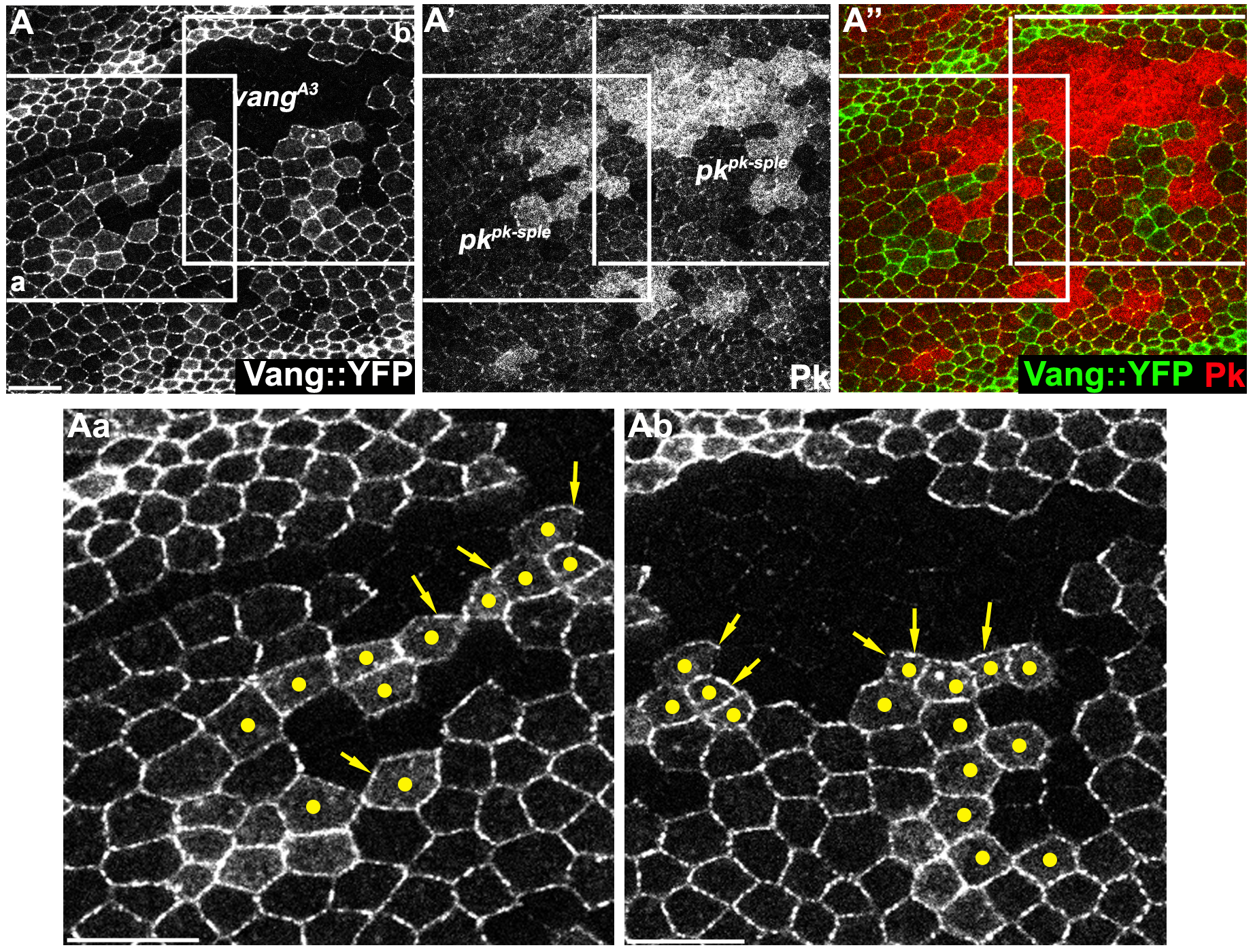

Supplement: S7 Fig — vang mutant clones in the presence of Fz recruit Vang from neighboring cells to the adjacent cell boundary, causing domineering non-autonomy. To assess whether Pk is required in the responding cell for Vang recruitment, we carried out a twinspot assay. FRT42D, vang A3 /FRT42D, pk pk-sple13, actP-vang::YFP (A) flies were used (vang mutant clones with Vang::YFP only in surrounding cells); some surrounding cells are wild-type, and others are pk mutant twin clones. Pk visualized by Pk staining (A’, red in A”). Yellow dots indicate pk mutant twin cells facing vang mutant clones (Aa and Ab: magnified images for squares in A). Vang::YFP is recruited to the adjacent membrane of cells abutting vang mutant cells regardless of whether they express pk (Aa and Ab; magnification of boxed regions in A; compare membranous Vang::YFP facing vang mutant cells in cells with and without yellow dots; yellow arrows indicate membranous Vang::YFP domains formed in pk mutant cells). 28hr APF. Scale bars: 10μm. Genotype: y, w, hsflp/+; FRT42D, vang A3 /FRT42D, pk pk-sple13, actP-vang::YFP. (TIF) [file pgen.1005259.s007.tif]
