# Supplementary material for: Clustering and Negative Feedback by Endocytosis in Planar Cell Polarity Signaling Is Modulated by Ubiquitinylation of Prickle
Source: PLoS Genet. 2015 May 21;11(5):e1005259. doi: 10.1371/journal.pgen.1005259 (PMC4440771; doi:10.1371/journal.pgen.1005259)
Supplement: S1 Table — Clonal mutation or overexpression effects of core PCP components on other core PCP factors at apical junctions, and hair polarities are compared to those resulting from clonal Cul1 complex mutation. (DOCX) [file pgen.1005259.s008.docx]

| **TableS1 Table. Apical patterns of core PCP factors and hair polarity in clonal wing tissues** |
| --- |

|  | Fmi | Fz | Dsh | Pk | Vang | Dgo | hair nonautonomy |
| --- | --- | --- | --- | --- | --- | --- | --- |
| *fmi* |  | down[[1](#_ENREF_1)] |  | down* | down*[[2](#_ENREF_2)] | down[[3](#_ENREF_3)] | none[[4](#_ENREF_4)] |
| *fz* | sym[[1](#_ENREF_1)] |  |  | down[[5](#_ENREF_5)] | down[[6](#_ENREF_6)] | down[[3](#_ENREF_3)] | toward[[7](#_ENREF_7)] |
| *dsh* | up[[2](#_ENREF_2),[6](#_ENREF_6)] | sym[[1](#_ENREF_1)] |  | down[[5](#_ENREF_5)] | sym[[6](#_ENREF_6)] |  | none[[8](#_ENREF_8)] |
| *pk-sple* | sym*[[6](#_ENREF_6)] | sym*[[6](#_ENREF_6)] |  |  | sym[[6](#_ENREF_6)] |  | none[[9](#_ENREF_9)] |
| *vang* | sym[[6](#_ENREF_6)] | down[[6](#_ENREF_6)] | down[[6](#_ENREF_6)] | up[[6](#_ENREF_6)] |  |  | away[[10](#_ENREF_10)] |
| *dgo* | sym[[3](#_ENREF_3)] |  |  |  |  |  |  |
|  | | | | | | | |
| *>>fmi* |  | up[[2](#_ENREF_2)] | up[[2](#_ENREF_2)] | down[[2](#_ENREF_2)] | down[[2](#_ENREF_2),[6](#_ENREF_6)] | down[[3](#_ENREF_3)] | toward[[5](#_ENREF_5)] |
| *>>fz* |  |  |  | up[[5](#_ENREF_5)] | sym[[6](#_ENREF_6)] | sym[[3](#_ENREF_3)] | away[[1](#_ENREF_1)] |
| *>>dsh* | up | sym |  |  | up[[6](#_ENREF_6)] |  | away[[11](#_ENREF_11)] |
| *>>pk* | up*[[5](#_ENREF_5),[6](#_ENREF_6)] | up*[[5](#_ENREF_5)] | up[[5](#_ENREF_5)] |  | up*[[6](#_ENREF_6)] |  | toward[[5](#_ENREF_5)] |
| *>>vang* | sym* | sym[[6](#_ENREF_6)] |  | sym[[6](#_ENREF_6)] |  |  | toward[[11](#_ENREF_11)] |
| *>>dgo* | up[[3](#_ENREF_3)] |  |  |  |  |  | away[[3](#_ENREF_3)] |
|  | | | | | | | |
| *cul1* complex -/- | up* | up* | up* | up* | up* |  | toward* |

sym = symmetric protein distribution

* this study

1. Strutt DI (2001) Asymmetric localization of Frizzled and the establishment of cell polarity in the *Drosophila* wing. Molecular Cell 7: 367-375.

2. Strutt H, Strutt D (2008) Differential stability of flamingo protein complexes underlies the establishment of planar polarity. Curr Biol 18: 1555-1564.

3. Feiguin F, Hannus M, Mlodzik M, Eaton S (2001) The ankyrin repeat protein Diego mediates Frizzled-dependent planar polarization. Dev Cell 1: 93-101.

4. Chae J, Kim MJ, Goo JH, Collier S, Gubb D, et al. (1999) The Drosophila tissue polarity gene starry night encodes a member of the protocadherin family. Development 126: 5421-5429.

5. Tree DR, Shulman JM, Rousset R, Scott MP, Gubb D, et al. (2002) Prickle mediates feedback amplification to generate asymmetric planar cell polarity signaling. Cell 109: 371-381.

6. Bastock R, Strutt H, Strutt D (2003) Strabismus is asymmetrically localised and binds to Prickle and Dishevelled during Drosophila planar polarity patterning. Development 130: 3007-3014.

7. Vinson CR, Adler PN (1987) Directional non-cell autonomy and the transmission of polarity information by the *frizzled* gene of *Drosophila*. Nature 329: 549-551.

8. Axelrod JD (2001) Unipolar membrane association of Dishevelled mediates Frizzled planar cell polarity signaling. Genes Dev 15: 1182-1187.

9. Adler PN, Taylor J, Charlton J (2000) The domineering non-autonomy of frizzled and van Gogh clones in the Drosophila wing is a consequence of a disruption in local signaling. Mech Dev 96: 197-207.

10. Taylor J, Abramova N, Charlton J, Adler PN (1998) Van Gogh: a new Drosophila tissue polarity gene. Genetics 150: 199-210.

11. Amonlirdviman K, Khare NA, Tree DR, Chen WS, Axelrod JD, et al. (2005) Mathematical modeling of planar cell polarity to understand domineering nonautonomy. Science 307: 423-426.
