# Supplementary material for: Clustering and Negative Feedback by Endocytosis in Planar Cell Polarity Signaling Is Modulated by Ubiquitinylation of Prickle
Source: PLoS Genet. 2015 May 21;11(5):e1005259. doi: 10.1371/journal.pgen.1005259 (PMC4440771; doi:10.1371/journal.pgen.1005259)
Supplement: S1 Text — Cloning procedures to generate C-terminal deleted pk (aa1-472) with N-terminal HA (YPYDVPDYA) tag is described. (DOCX) [file pgen.1005259.s009.docx]

**S1 Text**

**HA-tagged C-terminal deletion construct of *pk***

To generate the HA-tagged C-terminal deleted cDNA fragment of *pk* (*HA::pk^dC^*), RT-PCR was performed. Total RNAs (5µg) were isolated from *w1118* larvae using Trizol reagent (invitrogen) and the RT reaction was carried out using SuperScript^TM^ III first-strand synthesis system (invitrogen) to obtain the first DNA strand according to the manufacturer’s instructions. For the PCR reaction, primers, forward; 5’- gcggccgc ATG**TACCCATACGATGTTCCAGATTACGCT**ATGGATACCCCAAATCAAATGCCTG -3’ (NotI site at the 5’ of the start codon, start codon, and HA tag sequence (bold) added) and reverse; 5’- tctaga**TCA**CTCTCCTTTGCTGCAGGCGAT -3’ (*XbaI* site and the stop codon (bold) added) were used to amplify pk cDNA fragments encoding the amino acid sequence from 1 to 472. After the PCR reaction, the PCR products were digested with *NotI* and *XbaI* restriction enzymes and ligated into the pUASt vector.
